# Physiological changes after fluid bolus therapy in sepsis: a systematic review of contemporary data

**DOI:** 10.1186/s13054-014-0696-5

**Published:** 2014-12-27

**Authors:** Neil J Glassford, Glenn M Eastwood, Rinaldo Bellomo

**Affiliations:** Department of Intensive Care, Austin Hospital, Melbourne, Victoria 3084 Australia; Australian and New Zealand Intensive Care Research Centre, School of Public Health and Preventive Medicine, Monash University, Melbourne, Victoria 3004 Australia; School of Nursing and Midwifery, Faculty of Health, Deakin University, Burwood, Victoria 3125 Australia

## Abstract

Fluid bolus therapy (FBT) is a standard of care in the management of the septic, hypotensive, tachycardic and/or oliguric patient. However, contemporary evidence for FBT improving patient-centred outcomes is scant. Moreover, its physiological effects in contemporary ICU environments and populations are poorly understood. Using three electronic databases, we identified all studies describing FBT between January 2010 and December 2013. We found 33 studies describing 41 boluses. No randomised controlled trials compared FBT with alternative interventions, such as vasopressors. The median fluid bolus was 500 ml (range 100 to 1,000 ml) administered over 30 minutes (range 10 to 60 minutes) and the most commonly administered fluid was 0.9% sodium chloride solution. In 19 studies, a predetermined physiological trigger initiated FBT. Although 17 studies describe the temporal course of physiological changes after FBT in 31 patient groups, only three studies describe the physiological changes at 60 minutes, and only one study beyond this point. No studies related the physiological changes after FBT with clinically relevant outcomes. There is a clear need for at least obtaining randomised controlled evidence for the physiological effects of FBT in patients with severe sepsis and septic shock beyond the period immediately after its administration.

‘Just as water retains no shape, so in warfare there are no constant conditions’

Sun Tzu (‘The Art of War’)

**Electronic supplementary material:**

The online version of this article (doi:10.1186/s13054-014-0696-5) contains supplementary material, which is available to authorized users.

## Introduction

All critically ill patients receive intravenous (IV) fluids, which are given to maintain physiological homeostasis, or as a vehicle for drug administration, or as direct therapeutic administration to correct perceived haemodynamic instability [1-4]. In these situations, where there is a perceived reduction in venous return and cardiac output secondary to vasodilatation and/or hypovolaemia, using IV fluid to increase intravascular volume is believed to effectively compensate for these changes in vascular tone by increasing stroke volume in accordance with the Frank-Starling principle [5-10].

**Figure 1:**
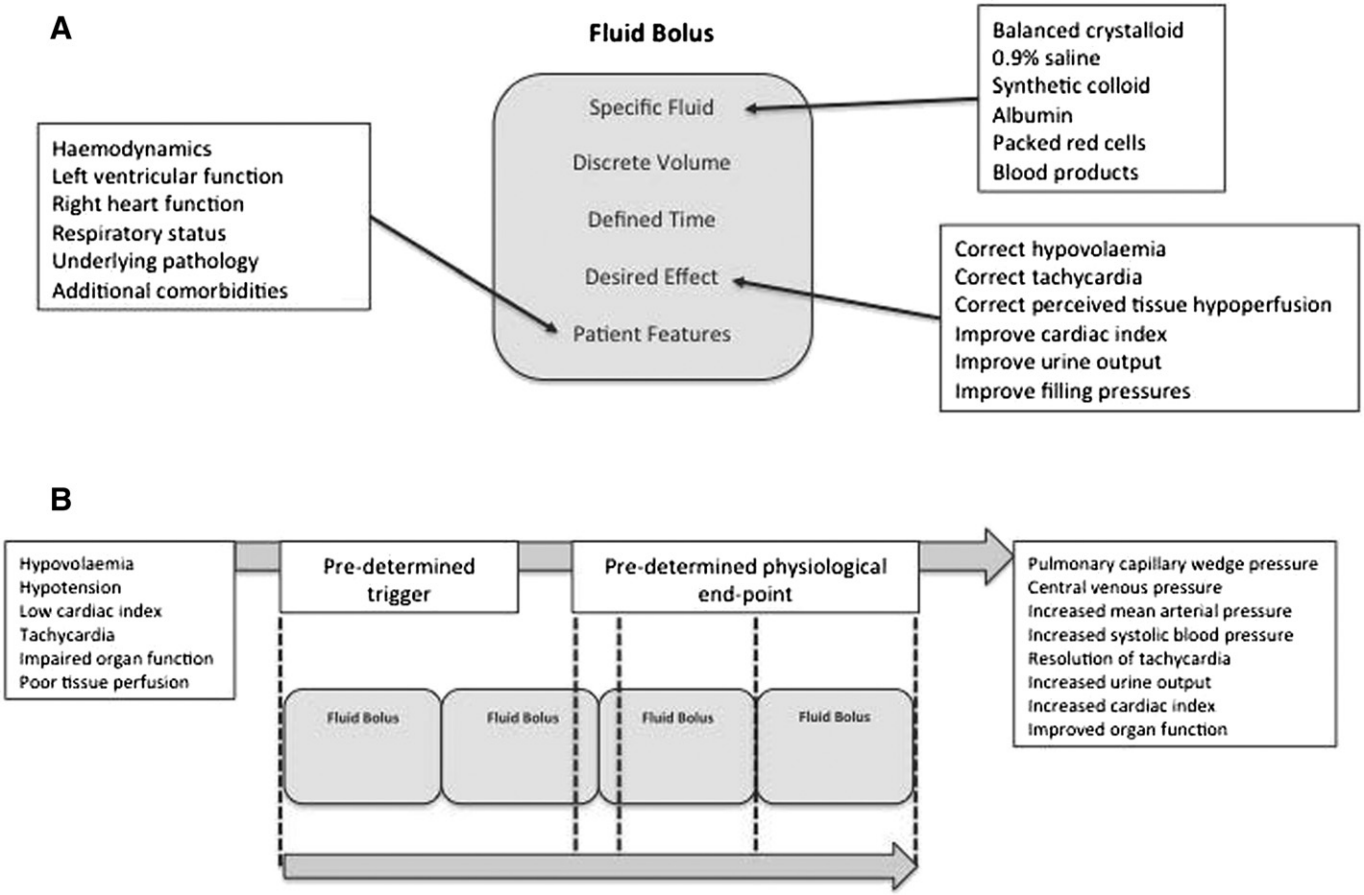
**Describing the concept of idealised fluid bolus therapy. (A)** Diagram describing the key criteria defining the concept of a fluid bolus. **(B)** Diagram describing the idealised concept of fluid bolus therapy in critical care, including purpose, triggers, end-points and purported physiological effects of such resuscitation.

Several mechanisms for delivering IV fluids, both diagnostically and therapeutically under such circumstances, have been described. These include Weil’s central venous pressure (CVP)-guided fluid challenge technique [10-13], the timed and rapid infusion methods favoured by Shoemaker [7,8,14-16] and, more recently, techniques involving echocardiographic or ultrasonographic assessment of fluid responsiveness following low-volume IV infusion [17]. However, the current standard of care in the management of septic, hypotensive, tachycardic and/or oliguric patients is fluid bolus therapy (FBT), where IV fluid is rapidly administered in discrete boluses [18-21]. While the ideal fluid bolus would be a discrete volume of a specific fluid administered at a specified rate, accounting for individual patient features and with a defined aim (Figure 1) [11], there is no current agreement regarding exactly what defines a fluid bolus. Moreover, although strong overall consensus regarding the importance of FBT exists [18-20], there appears to be little randomized controlled information on the magnitude and duration of its physiological effects, or on the direct positive impact of FBT on patient outcome in sepsis as an independent intervention [22].

In contrast, an expanding body of evidence suggests that FBT may contribute to a positive fluid balance, which, in turn, is independently associated with a variety of adverse outcomes in the critically ill [23-28]. Recent experimental evidence suggests rapid fluid infusion can also damage the endothelial glycocalyx [29,30], a structure already at risk in patients with sepsis [31], leading to endothelial disruption and organ dysfunction [32,33]. It appears that we need a better understanding of both the current evidence base for FBT and how best to apply it in the clinical setting [34,35].

Accordingly, we systematically reviewed the contemporary literature to determine current practice and to identify the independent effects of FBT on both physiological and patient-centred outcomes in the management of severe sepsis and septic shock in critical care practice.

## Methods

**Figure 2:**
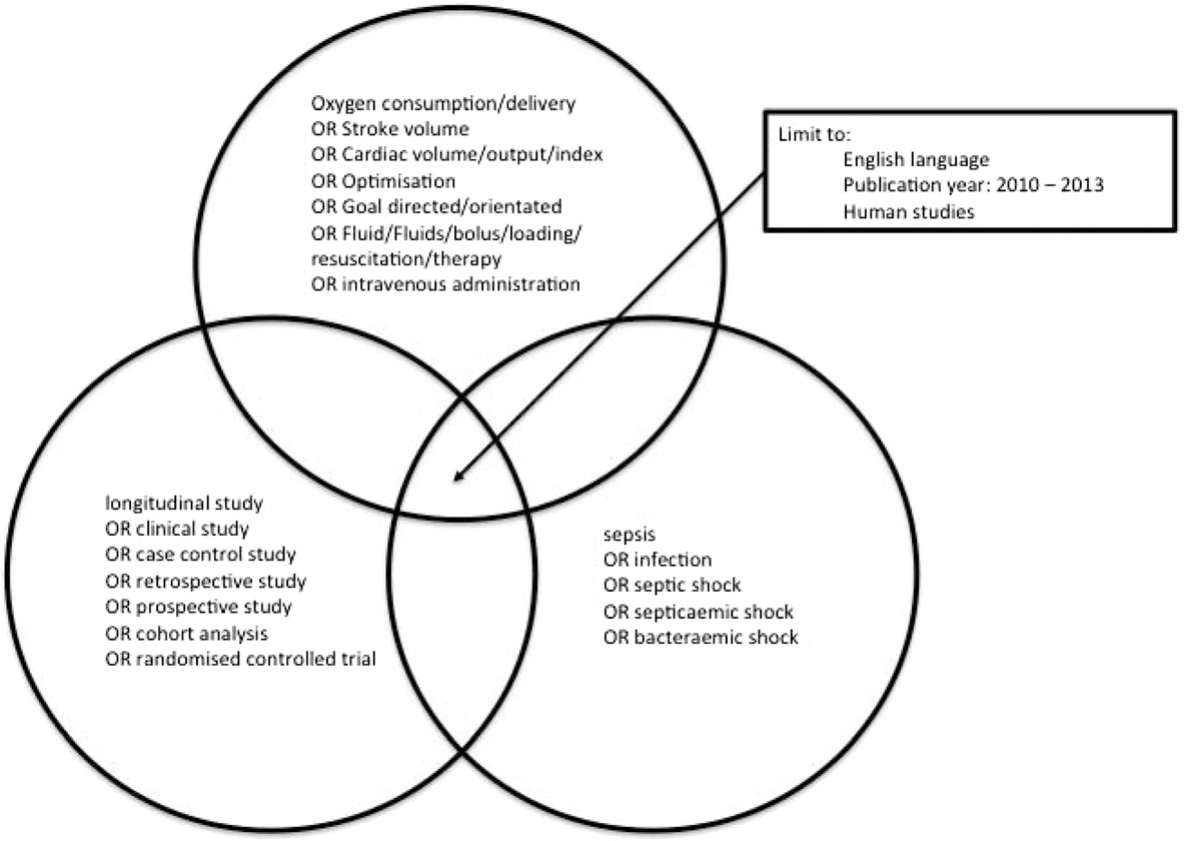
**Electronic search strategy.** Diagrammatic representation of the search strategy combining terms representing fluid resuscitation, sepsis and clinical studies, along with predetermined limitations.

We interrogated the MEDLINE, CENTRAL and EMBASE electronic reference databases using a combination of search terms (Figure 2). The reference lists of retrieved articles were examined for additional studies of potential relevance. The search was carried out in December 2013. To achieve contemporary relevance results were arbitrarily limited to this decade (2010 to 2013) and to English language studies in humans. Paediatric studies were excluded. This search defined a set of records of studies of fluid administration or haemodynamic optimization in patients with severe sepsis or septic shock.

The abstracts of these records were examined to identify those studies of potential relevance. These manuscripts were retrieved and examined manually in accordance with our inclusion criteria. The studies to be included in the review were checked to ensure they had not been retracted subsequent to their publication.

### Study inclusion criteria

#### Population of included studies

We considered clinical studies of any type describing a population of patients suffering from severe sepsis or septic shock. We also included those studies of shock or circulatory failure where either the majority of patients, or a defined subgroup of patients, had severe sepsis or septic shock.

#### Intervention - fluid bolus administration

For the purposes of this study a fluid bolus was a defined volume of a defined fluid administered over a defined time period. We recognised that most studies do not describe FBT in ideal terms (Figure 1) and therefore studies describing at least two of the three criteria were included in the review.

#### Comparator - alternatives to fluid administration

Any studies comparing FBT with the initiation of vasoactive medication, the increase of such medication or observation as an alternative to the administration of FBT were included in the review.

#### Between groups analysis

Where studies included in the review assigned patients to multiple treatment arms, each treatment group was treated as an individual group.

#### Outcome - physiological effects of bolus administration

Subsets of studies were selected from those describing FBT. The first included those reporting changes in cardiac output, heart rate, mean arterial pressure, central venous pressure, venous oxygen saturation, blood lactate concentration, urine output or haemoglobin concentration following FBT; for the purposes of inclusion, studies could describe changes in any or all of the haemodynamic parameters listed, but the direction, magnitude and duration of the change had to be extractable from tables or figures contained in the paper. The second group included those reporting non-physiological, patient-centred outcomes. Our primary outcome of interest was mortality at all reported time points. Secondary outcomes of interest included duration of ICU and hospital stay, duration of mechanical ventilation, and need for continuous renal replacement therapy (CRRT). We did not contact authors for additional information or individual patient data.

### Data collection

We collected data on study type, study setting and location, study population and the aims of the study. Due to our acceptance of multiple types of study, we chose not to adopt a methodological scoring system. We examined the definition of a fluid bolus in each study fulfilling our criteria and recorded the type and volume of fluid used, as well as the rate of administration. We identified the trigger and end-points for fluid bolus administration, the number of boluses administered and the use of red cell transfusions and vasoactive medication as part of the experimental protocol. We identified the demographic group in which subsequent observations were recorded. In those studies describing the physiological effects of bolus administration, we recorded the absolute change in cardiac output, heart rate, mean arterial pressure, venous oxygen saturation, blood lactate concentration, urine output and haemoglobin concentration. In those studies reporting patient-centred outcomes we recorded mortality at all reported time points, duration of ICU and hospital stay, duration of mechanical ventilation, and need for CRRT.

### Statistical analysis

We expected grossly heterogeneous results across different study types and study protocols. A meta-analysis approach could not be applied. Results are therefore presented as crude medians with full ranges. These exclude alternative units of measure, which are reported separately - for example, the median may be given in millilitres, followed by individual reporting of ml/kg.

## Results

### Electronic search

**Figure 3:**
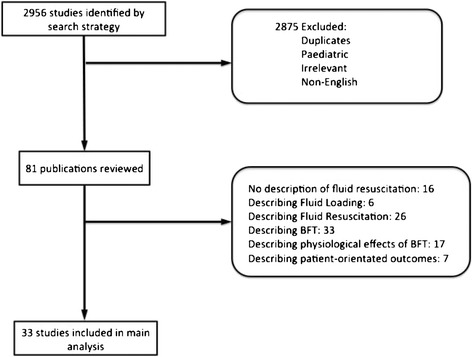
**Study selection.** Flow diagram of the study selection process and detailed description of study exclusions. FBT, fluid bolus therapy.

Our search strategy identified 2,956 articles over the period 2010 to 2013. Of these, 2,875 were excluded as duplicates, irrelevant, paediatric research or having been published in a language other than English. Of the 81 potentially relevant publications identified, 33 met our inclusion criteria (Figure 3) [36-68]. In total, 17 of these described the physiological changes occurring following FBT [36,39,40,45,46,48,50,53-55,57,59,60,62,63,65,66] and seven studies described patient-orientated outcome measures [37,42,43,49,58,59,64].

### Relevant contemporary studies

The study details, population, size and aims are presented in Table 1. We identified 22 prospective observational studies, four retrospective observational studies, two quasi-experimental studies, and five randomised controlled trials (RCTs). Of the five RCTs, none compared FBT with a control intervention; two actually reported the impact of blood volume analysis on protocolized resuscitation [64,67]; two compared hypertonic versus isotonic fluids [51,65]; and one actually compared two vasopressors and reported fluid data as an addendum [38]. Additional study data can be found in the electronic supplemental material (Additional file 1: Table S1).

**Table 1:** **Study settings, size, population and aims**

**First author**	**Journal**	**Year**	**Aims of study**	**Location**	**Institution(s)**	**Study type**	**Population size**
Bihari [36]	*Shock*	2013	Investigation of the use and effects of fluid boluses in septic patients following primary resuscitation	Australia	Single centre, academic ICU	Prospective observational study	50 patients with severe sepsis or septic shock
Castellanos-Ortega [37]	*Critical Care Medicine*	2010	Evaluation of the impact of a standardised EGDT response to sepsis	Spain	Single centre, academic ICU	Quasi-experimental study	480 patients with septic shock
De Backer [38]	*New England Journal of Medicine*	2010	Assessing the effect of noradrenaline as first-line vasopressor on mortality	Europe	8 centres, mixed ICUs	Randomised clinical trial	1,679 patients with shock requiring vaspressor therapy. 1,044 patients with sepsis
Dong [39]	*World Journal of Emergency Medicine*	2012	Investigating the relationship between stroke volume index and passive leg raising and fluid responsiveness	China	2 centres, general ICUs	Prospective observational study	32 mechanically ventilated patients with septic shock
Freitas [40]	*British Journal of Anaesthesia*	2013	Evaluation of the predictive value of automated PPV for fluid responsiveness in patients with sepsis and low tidal volumes	Brazil	Single centre, academic ICU	Prospective observational study	40 patients with low tidal volume ventilation and severe sepsis or septic shock requiring a fluid challenge
Gaieski [41]	*Critical Care Medicine*	2010	Evaluation of the impact of a standardised EGDT response to sepsis on time to antibiotic administration and survival	USA	Single centre, academic ICU	Retrospective observational study	261 patients with severe sepsis and septic shock undergoing EGDT
Hamzaoui [42]	*Critical Care*	2010	Evaluation of the cardiac consequences of early administration of noradrenaline	France	Single centre, academic ICU	Prospective observational study	105 patients with septic shock requiring vasopressor commencement following initial fluid resuscitation
Hanzelka [43]	*Supportive Care in Cancer*	2013	Evaluation of the impact of a standardised EGDT response to sepsis	USA	Single centre, academic ED	Retrospective observational study	200 patients with cancer and severe sepsis or septic shock presenting to ED
Jacob [44]	*Critical Care Medicine*	2012	Evaluation of the impact of early monitored sepsis management	Uganda	2 centres, medical/treatment centres	Prospective observational study	671 patients with severe sepsis presenting within office hours
Khwannimit [45]	*European Journal of Anaesthesiology*	2012	Comparing SVV by Vigileo with PPV by monitor to predict fluid responsiveness	Thailand	Single centre, academic ICU	Prospective observational study	42 patients with septic shock who were mechanically ventilated with tidal volumes >8 ml/kg requiring fluid resuscitation
Lakhal [46]	*Intensive Care Medicine*	2013	Identification of fluid responsiveness from IABP and NIBP	France	3 centres, academic ICU	Prospective observational study	130 patients with circulatory failure requiring a fluid challenge. 58 patients with septic shock
Lanspa [47]	*Journal of Critical Care*	2012	Assessment of CVP and shock index to predict haemodynamic response to volume expansion when compared with CVP alone	USA	Single centre, academic ICU	Prospective observational study	25 patients with septic shock over 14 years of age
Machare-Delgado [48]	*Journal of Intensive Care Medicine*	2011	Predicting fluid responsiveness by comparing SVV and inferior vena caval respiratory variation by ECHO during mechanical ventilation	USA	Single centre, medical academic ICU	Prospective observational study	25 mechanically ventilated vasopressor-dependent patients who required a fluid challenge. 22 patients with severe sepsis or septic shock
MacRedmond [49]	*Quality and Safety in Health Care*	2010	Evaluation of the impact of implementing a quality initiative on the management of severe sepsis and septic shock	Canada	Single centre, ICU	Quasi-experimental study	74 patients with severe sepsis or septic shock admitted via ED
Mahjoub [50]	*Intensive Care Medicine*	2012	Assessment of the impact of volume expansion on patients with left ventricular dysfunction	France	Single centre, academic ICU	Prospective observational study	83 mechanically ventilated patients with sepsis-induced circulatory failure
McIntyre [51]	*Journal of Critical Care*	2012	Feasibility study comparing the effects of 5% albumin versus 0.9% saline for resuscitation in septic shock	Canada	6 centres, academic ED and ICU	Randomised clinical trial	50 patients with refractory hypotension and sepsis
Monnet [52]	*Critical Care*	2010	Comparing haemodynamic changes induced by noradrenaline and volume expansion using Vigileo and PiCCO	France	Single centre, academic medical ICU	Prospective observational study	80 patients with sepsis-induced circulatory failure
Monnet [53]	*Critical Care Medicine*	2011	Assessing the effects of noradrenaline on haemodynamics in sepsis	France	Single centre, academic medical ICU	Prospective observational study	25 patients with sepsis-induced fluid-responsive acute circulatory failure with DBP <40 mmHg, or requiring noradrenaline
Monnet [54]	*Critical Care Medicine*	2013	Comparing ScvO_2_ and markers of anaerobic metabolism as predictors of unfavourable changes in oxygen extraction	France	Single centre, academic medical ICU	Prospective observational study	51 patients with acute circulatory failure undergoing transpulmonary thermodilution monitoring, 40 patients with septic shock
Monnet [55]	*Critical Care Medicine*	2011	Investigation of the utility of pulse pressure as a surrogate for changes in cardiac output	France	Single centre, academic medical ICU	Prospective observational study	373 patients with acute circulatory failure requiring a fluid challenge or the introduction or dose increase of noradrenaline. 338 patients with septic shock
O’Neill [56]	*Journal of Emergency Medicine*	2012	Evaluation of the most difficult elements of a SSC protocol to implement in a community-based ED	USA	Single centre, community ED	Retrospective observational study	79 with severe sepsis or septic shock remaining hypotensive following 2,000 ml of fluid resuscitation
Ospina-Tascon [57]	*Intensive Care Medicine*	2010	Evaluation of the effects of fluid administration on microcirculatory alterations in sepsis	Belgium	Single centre, academic ICU	Prospective observational study	60 patients with severe sepsis requiring fluid challenge. 37 within 24 hours of diagnosis, 23 after 48 hours
Patel [58]	*Annals of Pharmacotherapy*	2010	Investigation of the implementation and effects of introducing the SSC guidelines	USA	Single centre, community ICU	Prospective observational study	112 patients with sepsis or septic shock
Pierrakos [59]	*Intensive Care Medicine*	2012	Evaluation of the correlation between changes in MAP and CI following fluid challenge	Belgium	Single centre, academic ICU	Prospective observational study	51 patients with septic shock undergoing invasive haemodynamic monitoring and requiring a fluid challenge
Pottecher [60]	*Intensive Care Medicine*	2010	Assessment of sublingual microcirculatory changes in response to fluid challenge	France	2 centres, academic ED	Prospective observational study	25 mechanically ventilated patients with severe sepsis or septic shock within 24 hours of ICU admission demonstrating pre-load dependency
Sanchez [61]	*Anaesthesia and Intensive Care*	2011	Measuring the response to a fluid load in patients with and without septic shock	Spain	Single centre, academic ICU	Prospective observational study	32 patients requiring invasive monitoring. 18 patients with septic shock
Schnell [62]	*Critical Care Medicine*	2013	Assessment of the effects of a fluid challenge on Doppler-based renal resistive index in critically ill patients	France	3 centres, academic ICUs	Prospective observational study	35 mechanically ventilated patients with real-time cardiac monitoring requiring a fluid challenge. 30 patients with sepsis
Sturgess [63]	*Anaesthesia and Intensive Care*	2010	Comparison of aortic corrected flow time, BNP and CVP as predictors of fluid responsiveness	Australia	Single centre, private ICU	Prospective observational study	10 patients with septic shock requiring a fluid challenge
Trof [64]	*Critical Care Medicine*	2012	Comparison of volume-guided and pressure-guided hemodynamic management in shocked patients	Netherlands	2 centres, academic, ICU	Randomised clinical trial	120 patients with shock requiring invasive haemodynamic monitoring and >48 hours of ICU admission. 72 patients with sepsis
van Haren [65]	*Shock*	2012	Evaluation of the effects of hypertonic versus isotonic fluid administration in patients with septic shock	Netherlands	Single centre, academic ICU	Randomised clinical trial	24 patients with septic shock enrolled within 24 hours of admission
Wacharasint [66]	*Journal of the Medical Association of Thailand*	2012	Evaluation of the effectiveness of three dynamic measures of fluid responsiveness in septic shock patients	Thailand	Single centre, medical ICU	Prospective observational study	20 patients with sepsis and acute circulatory failure with invasive haemodynamic monitoring stable for 15 minutes prior to inclusion
Yu [67]	*Shock*	2011	Evaluation of the effects of blood volume analysis compared with pulmonary artery catheter monitoring	North America	Single centre, academic ICU	Randomised clinical trial	100 patients requiring resuscitation for shock. 69 patients with severe sepsis or septic shock
Zhang [68]	*Journal of Critical Care*	2012	Investigation of the association between plasma protein levels and subsequent pulmonary oedema	China	Single centre, academic ICU	Retrospective observational study	62 patients with sepsis undergoing transpulmonary thermodilution assessment requiring fluid

BNP, B-type natriuretic peptide; CI, cardiac index; CVP, central venous pressure; DBP, diastolic blood pressure; ECHO, echocardiogram; ED, Emergency Department; EGDT, early goal directed therapy; IABP, intra-arterial blood pressure; MAP, mean arterial blood pressure; NIBP, non-invasive blood pressure; PiCCO, pulse contour cardiac output monitoring; PPV, pulse pressure variation; ScvO_2_, central venous oxygen saturation; SSC, Surviving Sepsis Campaign; SVV, stroke volume variation.

### Pre-fluid bolus therapy fluid administration

Fluid resuscitation prior to study recruitment and FBT was described in 10 studies. In the five studies describing finite volumes of resuscitation fluid, the median volume administered was 2,200 ml (range 1,000 to 5,060 ml) [38,47,51,53,58]. The five remaining studies reported weight-dependent volumes of between 20 and 30 ml/kg of resuscitation (Table 2) [41,43,49,56,57].

**Table 2:** **Description of fluid boluses, triggers, physiological end-points and primary confounders**

**First author**	**Year**	**Initial resuscitation**	**Bolus fluid type**	**Bolus fluid volume (ml)**	**Bolus fluid rate (minutes)**	**Physiological trigger for fluid administration**	**Physiological end-point for fluid administration**	**Number of boluses administered**	**Vasoactive administration?**	**Packed red cell transfusion?**
Bihari [36]	2013	Undefined	4% albumin	750	<30	Clinician defined	Clinician defined	2	Yes	Not described
			Packed red cells							
			20% albumin							
			Fresh frozen plasma							
			4% gelatin							
			0.9% saline							
Castellanos-Ortega [37]	2010	Undefined	Crystalloid	1,000	30	Hypotension	CVP ≥8 mmHg, MAP ≥65 mmHg, ScvO_2_ ≥ 70%	Not described	Yes	Not described
			Colloid	500						
De Backer [38]	2010	500 ml colloid or 1,000 ml crystalloid	Crystalloid	1,000	Not defined	MAP <70 mmHg; SBP <100 mmHg, altered mental state; mottled skin; oliguria >1 hour, hyperlactataemia	Not described	Not described	Yes	Not described
			Colloid	500						
Dong [39]	2012	Undefined	6% HES	500	30	SBP <90 mmHg or >40 mmHg drop or need for vasopressors, oliguria >1 hour; mottled skin; HR >100 bpm	End of infusion.	1	Not described	Not described
Freitas [40]	2012	Undefined	6% HES	7 ml/kg (max 500)	30	Clinician defined	End of infusion	1	Yes	No
Gaieski [41]	2010	20-30 ml/kg	0.9% saline	500	15-20	CVP <8 mmHg	CVP >8 mmHg	Not described	Yes	Yes
Hamzaoui [42]	2010	Undefined	0.9% saline	1,000	Not defined	Undefined	Not described	Not described	Yes	Not described
Hanzelka [43]	2013	20 ml/kg	Undefined	1,000	60	Severe sepsis	SBP >90 mmHg, MAP <65 mmHg	Not described	Yes	No
				500	30					
Jacob [44]	2012	Undefined	0.9% saline	1,000	60	SBP <100 mmHg or hyperlactataemia	SBP increased by 10 mmHg for 2 consecutive hours to >90 mmHg	Up to 10	No	Not described
				500	30					
Khwannimit [45]	2012	Undefined	6% HES	500	30	Clinician defined	End of infusion	1	Yes	Not described
Lakhal [46]	2013	Undefined	4% gelatin	500	30	One or more of SBP <90 mmHg, MAP <65 mmHg , requiring vasoactive medication, oliguria, skin mottling, hyperlactataemia	End of infusion	1	Yes	Not described
Lanspa [47]	2012	5,060 ml	Crystalloid (or equivalent colloid)	20 ml/kg	<20	Clinician defined	End of infusion	1.36	Yes	Yes
Machare-Delgado [48]	2011	Undefined	0.9% saline	500	10	Clinician defined	End of infusion	1	Not described	No
MacRedmond [49]	2010	25 ml/kg	0.9% saline	500	<15	MAP <65 mmHg	CVP 8-12; MAP >65 mmHg; ScvO_2_ > 70%	Not described	Yes	Yes
Mahjoub [50]	2013	Undefined	0.9% saline	500	20	SBP <90 mmHg and/or need for vasoactive drugs and/or persistent lactic acidosis	End of infusion	1	Yes	Not described
McIntyre [51]	2012	2,400 ml	0.9% saline or 4% albumin	500	STAT	Undefined	Not described	6	Yes	Not described
Monnet [52]	2010	Undefined	0.9% saline	500	30	SBP <90 mmHg, SBP drop >50 mmHg if HT, and one or more of HR >100, skin mottling or oliguria	End of infusion	1	Yes	Not described
Monnet [53]	2011	2,200 ml	0.9% saline	500	10	SBP <90 mmHg, SBP drop >50 mmHg if HT, and one or more of HR >100, skin mottling or oliguria	End of infusion	1	Yes	Not described
Monnet [54]	2013	Undefined	0.9% saline	500	30	SBP <90 mmHg, SBP drop >50 mmHg if HT, and one or more of HR >100, skin mottling or oliguria	End of infusion	1	Yes	Yes
Monnet [55]	2011	Undefined	0.9% saline	500	20	SBP <90 mmHg, SBP drop >50 mmHg if HT, and one or more of HR >100, skin mottling or oliguria	End of infusion	1	Yes	Not described
O’Neill [56]	2012	20 ml/kg	0.9% saline	500	15	CVP <8 mmHg; MAP <65 mmHg; ScvO_2_ < 70%	CVP 8-12; MAP >65 mmHg; ScvO_2_ > 70%	0.68	Yes	Not described
Ospina-Tascon [57]	2010	Undefined	CSL	1,000	30	MAP <65 mmHg	End of infusion	1	Yes	Not described
			4% albumin	400						
Patel [58]	2010	2,000 ml	Normal saline	Undefined	30	SBP <90 mmHg; MAP <65 mmHg	Not described	1	Yes	Not described
Pierrakos [59]	2012	Undefined	CSL	100	30	Clinician defined	End of infusion	1	Yes	Not described
			6% HES	500						
Pottecher [60]	2010	Undefined	HES 6% or 0.9% saline	500	30	MAP <65 mmHg, skin mottling or oliguria	End of infusion	1	Yes	Not described
Sanchez [61]	2011	Undefined	Crystalloid	1,000	Undefined	Hypotension with perfusion abnormalities		Not described	Yes	No
			Colloid	500			ITBVI >900 ml/ml or EVLWI >10 ml/kg			
Schnell [62]	2013	Undefined	0.9% saline	500	15-30	Clinician defined	End of infusion	1	Yes	Not described
Sturgess [63]	2010	Undefined	4% albumin	250	15	Clinician defined	End of infusion	1	Yes	No
Trof [64]	2012	Undefined	HES or 4% gelatin	250-500	30	EVLWI <10 ml/kg or >10 ml/kg with GEDVI <850 ml/m^2^; PAOP >18 mmHg; MAP <65 mmHg, HR >100, SvO_2_ < 65% or ScvO_2_ < 70%; oliguria; peripheral perfusion deficits, hyperlactatemia	MAP >65 mmHg, ScvO_2_ > 70%, lactate clearance, diuresis >0.5 ml/kg/hour, restoration of peripheral perfusion deficits	3.48	Yes	Not described
van Haren [65]	2012	Undefined	6% HES in 0.9% saline	500	15	Septic shock	End of infusion	1	Yes	Not described
				250	15					
			6% HES in 7.2% saline							
Wacharasint [66]	2013	Undefined	HES 6%	500	30	SBP <90 mmHg or requirement for vasopressors	End of infusion	1	Yes	Not described
Yu [67]	2011	30 ml/kg in 1,000 ml increments	Crystalloid or colloid	250-500	Undefined	PAOP <12 mmHg or 12-17 mmHg with	SBP >100 mmHg, HR <100 bpm, UO >0.5 ml/kg/hour, lactate clearance, SmvO_2_ > 70%	Not described	Not described	Yes
						SBP <100; HR >100 bpm UO <0.5 ml/kg/hour; hyperlactataemia; SvO_2_ > 70% or equivalent blood volume goals				
Zhang [68]	2012	Undefined	Crystalloid or colloid	250-500	30	SBP <90 mmHg; HR >100 bpm; GEDVI <700 ml/m^2^; CVP <12 mmHg (PEEP dependent)	Pre-defined rise in CVP	Not described	Yes	Not described

CSL, compound sodium lactate solution; CVP, central venous pressure; EVLWI, extra-vascular lung water index; HES, hydroxyethyl starch; HR, heart rate; HT, hypertensive; GEDVI, global end diastolic volume index; ITBVI, intrathoracic blood volume index; MAP, mean arterial blood pressure; PAOP, pulmonary artery occlusion pressure; PEEP, positive end-expiratory pressure; SBP, systolic blood pressure; ScvO_2_, central venous oxygen saturation; SmvO_2_, mixed venous oxygen saturations; STAT, statim/immediately; SvO_2_, venous oxygen saturation; UO, urine output.

### Initiation and cessation of fluid bolus therapy

Across the 33 studies, 19 predetermined clinical or physiological features triggered FBT. In the remaining 14 studies, FBT was triggered by clinical judgment in eight, by ‘hypotension’ in two, simply by the diagnosis of severe sepsis or septic shock in two, and remained unspecified in two (Table 2).

In the majority of studies (18 of 33) FBT ceased at the end of the bolus in question; 10 studies used predetermined immediate changes in physiological variables as end-points; four studies did not define the physiological end-points of fluid resuscitation (Table 2).

### Defining fluid bolus therapy

Overall, 41 forms of FBT were described, fully or in part, in 33 studies. They are presented in Table 2. In 20 studies, the fluid type was fixed; in 13 more than one fluid type was used. In six studies the fluid type was not identified beyond the generic ‘crystalloid or colloid’. The fluid most commonly used as a bolus was 0.9% saline (17 studies), followed by 6% hydroxyethyl starch (eight studies). On the other hand, 4% albumin was used in only four studies [38,53,59,65], 4% gelatin in only three [38,48,66], physiological lactated solutions in only two [59,61], and 20% albumin and blood products in only one [38].

The median amount of fluid administered as a finite volume was 500 ml (range 100 to 1,000 ml). However, 20 ml/kg and 7 ml/kg were individually reported as weight-dependent boluses. The median number of boluses (24 studies) was 1 (range 0.68 to 10). Rates of administration were defined for 31 of 41 boluses with a median rate of 30 minutes (range 10 to 60 minutes).

### Haemodynamic changes after fluid bolus therapy

#### Comparing different interventions

No RCTs compared the haemodynamic changes induced by FBT with ‘observation’ or ‘vasopressor administration’ or ‘inotropic drug administration’ or ‘continuous low dose IV fluid infusion’ or any combination of the above. The only study comparing FBT with an alternative intervention was a single, non-randomized, prospective, observational study that compared acute circulatory failure patients treated with FBT (500 ml of saline) or with increased norepinephrine dose according to clinician preference [55]. The two groups had clearly different baseline characteristics and were not directly compared.

### Temporal trends in physiological changes following fluid bolus therapy

The temporal change in physiological parameters following FBT is described in 31 different groups across 17 studies (Table 3).

**Table 3:** **Physiological effects grouped by measurement time**

**First author**	**Fluid given**	**Group**	**Time from completion of fluid administration until physiological measurement (minutes)**	**Measure of central tendency**	**Change in cardiac output estimation**	**Change in heart rate (bpm)**	**Change in mean arterial pressure (mmHg))**	**Change in central venous pressure (mmHg)**	**Change in venous oxygen saturation (%)**	**Change in blood lactate concentration (mmol/l)**	**Change in urine output**	**Change in haemoglobin concentration (g/L)**
**Haemodynamic indices measured immediately following fluid bolus administration**												
Machare-Delgado [48]	500 ml of 0.9% saline over 10 minutes	Responders: >10% SVI increase	0	Mean	+3.99 ml/m^2^/beat							
	500 ml of 0.9% saline over 10 minutes	Non-responders: >10% SVI increase	0	Mean	+0.57 ml/m^2^/beat							
Dong [39]	500 ml of 6% HES over 30 minutes	Responders: >15% SVI increase	0	Mean	+600 ml/min/m^2^	-1.5	+15.2	+3.2				
	500 ml of 6% HES over 30 minutes	Non-responders: <15% SVI increase	0	Mean	+300 ml/min/m^2^	-1.2	+4.8	+2.3				
Khwannimit [45]	500 ml of 6% HES over 30 minutes	Responders: >15% SVI increase	0	Mean	+1300 ml/min/m^2^	-3.3	+9.5	+3.4				
	500 ml of 6% HES over 30 minutes	Non-responders: <15% SVI increase	0	Mean	+200 ml/min/m^2^	-0.9	+3.9	+5.2				
Lakhal [46]	500 ml of 4% gelatin over 30 minutes	Responders: >15% SVI increase	0	Mean	+900 ml/min/m^2^	-6	+14	+3				
	500 ml of 4% gelatin over 30 minutes	Non-responders: <15% SVI increase	0	Mean	+0 ml/min/m^2^	-3	+7	+4.5				
Mahjoub [50]	500 ml of 0.9% saline over 20 minutes	Responders: >10% SV increase	0	Mean	+1,000 ml/min	-4	+7	+2.6				
	500 ml of 0.9% saline over 20 minutes	Non-responders: >10% SV increase	0	Mean	+300 ml/min	-3	+1	+2.9				
Monnet [53]	500 ml of 0.9% saline over 10 minutes	All patients	0	Mean	+800 ml/min/m^2^	-7	+8	+5				
Monnet [55]	500 ml of 0.9% saline over 20 minutes	Responders: >15% CI increase	0	Mean	+800 ml/min/m^2^	-2	+11					
	500 ml of 0.9% saline over 20 minutes	Non-responders: <15% increase in CI	0	Mean	+200 ml/min/m^2^	-2	+4					
Monnet [54]	500 ml of 0.9% saline over 30 minutes	Responders: >15% VO_2_ increase	0	Mean	+1,000 ml/min/m^2^	-2	+7		+1%	-1.9		-7
	500 ml of 0.9% saline over 30 minutes	Non-responders: <15% increase in VO_2_	0	Mean	+1,000 ml/min/m^2^	+0	+13		+7%	-0.3		-6
Schnell [62]	500 ml of 0.9% saline over 15-30 minutes	Responders: >10% increase in aortic blood flow	0	Median	+20 ml/beat	-10	+7					
	500 ml of 0.9% saline over 15-30 minutes	Non-responders: <10% increase in aortic blood flow	0	Median	+8 ml/beat	-1	+6					
Sturgess [63]	250 ml of 4% albumin over 15 minutes	All patients	0	Mean	+7.5% ml/beat							
**Haemodynamic indices measured 30 minutes after fluid bolus administration**												
Freitas [40]	7 ml/kg, maximum 500 ml, of 6% HES over 30 minutes	Responders: >15% CO increase	30	Mean	+2,100 ml/min	-2	+11	+3	+8%	-0.1		
	7 ml/kg, maximum 500 ml, of 6% HES over 30 minutes	Non-responders: <15% increase in CO	30	Mean	+200 ml/min	+0	+8	+5	-3.5%	-0.2		
Pierrakos [59]	500 ml of 6% HES or 1,000 ml of CSL over 30 minutes	Responders: >10% increase in CI	30	Mean	+600 ml/min/m^2^	-4	+8	+3	+3%			
	500 ml of 6% HES or 1,000 ml of CSL over 30 minutes	Non-responders: <10% increase in CI	30	Mean	+0 ml/min/m^2^	-4	+3	+2	+0%			
Pottecher [60]	Up to 500 ml of 6% HES or 0.9% saline over 30 minutes	All patients	30	Mean	+1,400 ml/min	-2	+7					
Wacharasint [66]	500 ml of 6% HES over 30 minutes	All patients	30	Mean	+470 ml/min/m^2^	+0.3	+9.2	+5.25				
van Haren [65]	250 ml of 6% HES in 7.2% saline over 15 minutes	Hypertonic bolus	30	Mean	+300 ml/min/m^2^	-11	+4	+2		-0.2		-8
	500 ml of 6% HES in 0.9% saline over 15 minutes	Isotonic bolus	30	Mean	-400 ml/min/m^2^	-1	+5	+4		-0.1		-9
**Haemodynamic indices measured 60 minutes after fluid bolus administration**												
Bihari [36]	500-750 ml of 4% albumin, blood, 20% albumin FFP, 0.9% saline, 4% gelatin or platelets administered over less than 30 minutes	All patients	60	Median		+0	+2	+2	+0.4%	-0.2	No change	-6
Ospina-Tascon [57]	400 ml of 4% albumin or 1,000 ml of CSL over 30 minutes	Patients with early sepsis	60	Median	+300 ml/min/m^2^	+2	+2	+3	+2%	-0.2		
	400 ml of 4% albumin or 1,000 ml of CSL over 30 minutes	Patients with late sepsis	60	Median	+300 ml/min/m^2^	-9	+7	+1	+1%	+0.1		
van Haren [65]	250 ml of 6% HES in 7.2% saline over 15 minutes	Hypertonic bolus	60	Mean	+400 ml/min/m^2^	-11	+6	+1		-0.3		-9
	500 ml of 6% HES in 0.9% saline over 15 minutes	Isotonic bolus	60	Mean	-300 ml/min/m^2^	-1	+3	+3		-0.1		-12
**Haemodynamic indices measured greater than 60 minutes after fluid bolus administration**												
van Haren [65]	250 ml of 6% HES in 7.2% saline over 15 minutes	Hypertonic bolus	120	Mean	+300 ml/ml/m^2^	-7	+7	+2		0.0	+13	-6
	500 ml of 6% HES in 0.9% saline over 15 minutes	Isotonic bolus	120	Mean	-300 ml/min/m^2^	+0	+1	+2		-0.3	-30	-9
	250 ml of 6% HES in 7.2% saline over 15 minutes	Hypertonic bolus	180	Mean	+100 ml/min/m^2^	-3	+6	+3		-0.3		-9
	500 ml of 6% HES in 0.9% saline over 15 minutes	Isotonic bolus	180	Mean	+0 ml/min/m^2^	+3	+5	+3		-0.2		-6
	250 ml of 6% HES in 7.2% saline over 15 minutes	Hypertonic bolus	240	Mean	+100 ml/min/m^2^	+1	+3	+3		-0.3	-3	-8
	500 ml of 6% HES in 0.9% saline over 15 minutes	Isotonic bolus	240	Mean	-200 ml/min/m^2^	+3	+0	+3		-0.2	-40	-4

CI, cardiac index; CO, cardiac output; CSL, compound sodium lactate; FFP, fresh frozen plasma; HES, hydroxyethyl starch; SVI, stroke volume index; VO_2_, oxygen delivery.

#### Immediately post-infusion

Ten studies reported the physiological state after bolus administration in 18 groups immediately post-administration. In the six studies describing changes in cardiac index immediately post-FBT, cardiac index increased by a median of 800 ml/minute/m^2^ (range 0 to 1,300 ml/minute/m^2^). The median reduction in heart rate at the end of a fluid bolus (eight studies) was 2 bpm (range 10 to 0 bpm reduction) and the median increase in mean arterial pressure (eight studies) was 7 mmHg (range 1 to 15.2 mmHg). The median increase in CVP across five studies was 3.2 mmHg (range 2.3 to 5.2 mmHg). Only a single study reported the effect on venous oxygen saturation, blood lactate concentration or haemoglobin concentration. No study reported the effect on urine output.

#### Thirty minutes post-administration

Five studies reported the physiological effects of FBT 30 minutes after administration. Cardiac index increased by a median of 300 ml/minute/m^2^ (range -400 to 600 ml/minute/m^2^) in three studies. The median reduction in heart rate (five studies) was 2 bpm (range 11 bpm reduction to 0.3 bpm increase) and the median increase in mean arterial pressure (five studies) was 7.5 mmHg (range 3 to 11 mmHg). The median increase in CVP across four studies was 3 mmHg (range 2 to 5.25 mmHg). There was a median increase in central venous saturation of 2% (range 4% reduction to 8% increase) across two studies. Changes in other indices are reported in Table 3.

#### Sixty minutes post-administration

**Figure 4:**
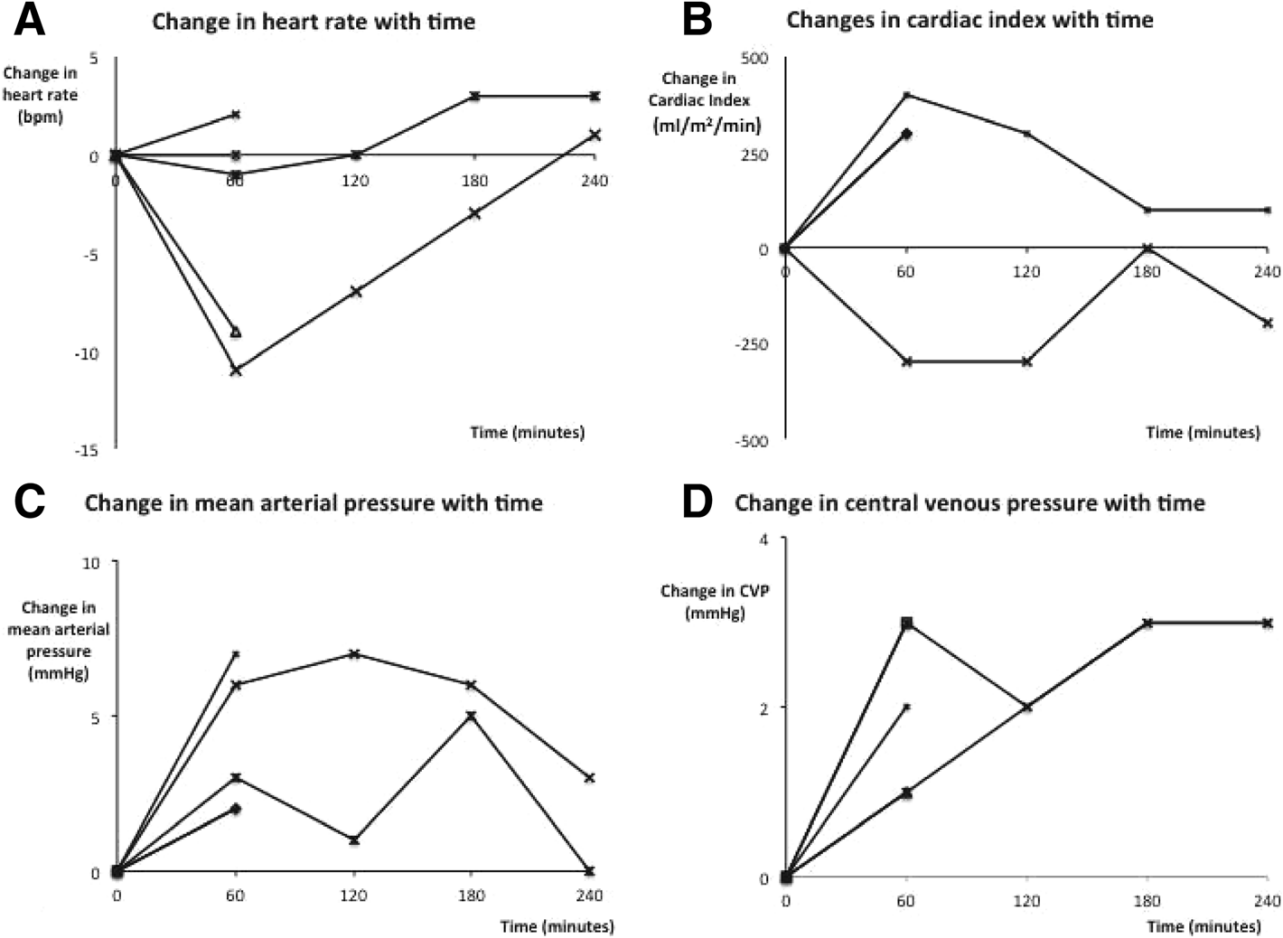
**Physiological effects of fluid bolus therapy over time.** Multi-panel figure of the haemodynamic effects of fluid bolus therapy (FBT) as reported in studies with observation periods of 60 minutes or more. **(A)** Changes in heart rate over time. **(B)** Changes in cardiac index over time. **(C)** Changes in mean arterial pressure over time. **(D)** Changes in central venous pressure (CVP) over time. Each solid black line represents a patient group and the average physiological response to FBT over the observation period. Lines terminate when measurements were discontinued in the study from which the group was taken.

Only three studies reported the physiological effects of FBT 60 minutes after administration (Figure 4) [36,57,65]. Cardiac index increased by a median of 300 ml/minute/m^2^ (range -300 to 400 ml/minute/m^2^) in two studies. The median reduction in heart rate 60 minutes after a fluid bolus (three studies) was 1 bpm (range 11 bpm reduction to 2 bpm increase) and the median increase in mean arterial pressure (three studies) was 3 mmHg (range 2 to 7 mmHg). The median increase in CVP across three studies was 2 mmHg (range 1 to 3 mmHg). There was a median increase in central venous saturation of 1% (range 0.4% to 2% increase) across two studies.

#### Beyond 1 hour post-fluid bolus therapy

Only one study reported the effects of BFT at 120, 180 and 240 minutes after administration (Figure 4) [65].

### Comparing responders and non-responders

Overall, 10 studies compared the physiological responses to FBT administration between groups defined by changes in a physiological variable. Patients were defined as either responders or non-responders depending on the response exhibited. Different variables are used in different studies: stroke volume index (five studies), cardiac index or output (three studies), increase in oxygen consumption (one study) or aortic blood flow rate (one study). All reported changes only within 30 minutes of FBT completion (Additional file 1: Table S2).

In the six studies describing changes in cardiac index, cardiac index increased by a median of 850 ml/minute/m^2^ (range 600 to 1,300 ml/minute/m^2^) in fluid responders compared with 200 ml/minute/m^2^ (range 0 to 1,000 ml/minute/m^2^) in non-responders. The median increase in mean arterial pressure (10 studies) in responders was 9.5 mmHg (range 7 to 15.2 mmHg) versus 4.8 mmHg (range 1 to 13 mmHg) in non-responders. Similarly, the median increase in central venous pressure (six studies) was 3 mmHg (range 2.6 to 3.4 mmHg) in responders versus 3.7 mmHg (range 2 to 5.2 mmHg) in non-responders. The median decrease in heart rate (nine studies) was 3.3 bpm in responders (range 1.5 to 10 bpm decrease) and 1.2 bpm in non-responders (range 0 to 4 bpm decrease). Information on changes in venous oxygen saturation, blood lactate concentration, and blood haemoglobin concentration in the few studies reporting such data are presented in Additional file 1: Table S2.

### Additional comparisons

The physiological effects of FBT grouped by speed of FBT delivery (Additional file 1: Table S3) and by class of fluid administered (Additional file 1: Table S4) have also been presented. There is no consistent pattern demonstrated across or between groups.

### Relationship between physiological changes after fluid bolus therapy and clinical outcome

Overall, seven studies described clinically orientated outcomes [37,43,44,49,58,59,64]. All reported the effects of complex interventions, such as early goal-directed therapy. No studies examined the relationship between FBT and outcome directly (Tables 4 and 5).

**Table 4:** **Clinically orientated primary outcomes**

**First author**	**Journal**	**Year**	**Control group**	**ICU mortality**	**Hospital mortality**	**Other**	**Intervention group**	**ICU mortality**	**Hospital mortality**	**Other**
**MacRedmond** **[** 49 **]**	*Quality and Safety in Health Care*	2010	Before protocolised resuscitation	19/37			After protocolised resuscitation	10/37		
**Pierrakos** **[** 59 **]**	*Intensive Care Medicine*	2012	Responders (>10% increase in CI)	13/25			Non-responders (<10% increase in CI)	11/26		
**Patel** **[** 58 **]**	*Annals of Pharmacotherapy*	2010	Pre-intervention		32/53		Post-intervention, significantly more fluid and less vasoactives		12/59	
**Castellanos-Ortega** **[** 37 **]**	*Critical Care Medicine*	2010	Pre-intervention	51/96	55/96		Post-intervention, significantly more fluid	117/384	144/384	
**Trof** **[** 64 **]**	*Critical Care Medicine*	2012	Pulmonary artery catheter-guided resuscitation	13/34	15/34		Transpulmonary thermodilution-guided resuscitation	17/38	21/38	
**Hanzelka** **[** 43 **]**	*Supportive Care in Cancer*	2013	Pre-intervention			28-day: 38/100	Post-intervention, significantly quicker resuscitation			28-day: 20/100
**Jacob** **[** 44 **]**	*Critical Care Medicine*	2012	Pre-intervention			30-day: 126/245	Post-intervention, significantly quicker resuscitation with significantly larger volumes of fluid at 6 and 24 hours			30-day: 257/426

CI, cardiac index.

**Table 5:** **Clinically orientated secondary outcomes**

**First author**	**Journal**	**Year**	**Control group**	**LOS in ICU (days)**	**LOS in hospital (days)**	**MV (days)**	**CRRT**	**Intervention group**	**LOS in ICU (days)**	**LOS in hospital (days)**	**MV (days)**	**CRRT**
**MacRedmond** **[** 49 **]**	*Quality and Safety in Health Care*	2010	Before protocolised resuscitation	8				After protocolised resuscitation	7			
**Castellanos-Ortega** **[** 37 **]**	*Critical Care Medicine*	2010	Pre-intervention	9.9	26.5			Intervention group, significantly more receive fluid	9.1	30.6		
**Hanzelka** **[** 43 **]**	*Supportive Care in Cancer*	2013	Pre-intervention	5.1	10.3			Post-intervention, significantly quicker resuscitation	2.5	8.1		
**Trof** **[** 64 **]**	*Critical Care Medicine*	2012	Pulmonary artery catheter-guided resuscitation	15	25	13		Transpulmonary thermodilution-guided resuscitation	11	27	10	
**Patel** **[** 58 **]**	*Annals of Pharmacotherapy*	2010	Pre-intervention	6	9.5	7.5	8/53	Post-intervention, significantly more fluid and less vasoactives	5	9	7	0/59

CRRT, continuous renal replacement therapy; LOS, length of stay; MV, mechanical ventilation.

## Discussion

We examined the contemporary literature on FBT in severe sepsis and septic shock and identified 33 original studies describing the characteristics of a fluid bolus, 17 of which also describe the associated physiological changes. We found heterogeneity of triggers, amount, fluid choice and speed of delivery for FBT, which was administered to achieve heterogeneous physiological targets. We similarly found heterogeneity of physiological changes after FBT. In addition, no RCTs compared FBT with an alternative intervention. Finally, no study related physiological changes after FBT to clinically relevant outcomes.

FBT is a widespread intervention in the management of the critically ill septic patient, despite lack of a consistent definition or use of terminology. Our study demonstrates that no contemporary RCTs exist that compare FBT with alternative interventions. The only study comparing FBT to an alternative intervention was a single, non-randomized, prospective, observational study that compared acute circulatory failure patients treated with FBT (500 ml of saline) or with increased norepinephrine dose according to clinician preference. The two groups had clearly different baseline characteristics and were not directly compared [55]. Alternative interventions to FBT may include a diagnostic low-volume FBT [17], classic fluid challenge [11,12], low-volume FBT and low-dose vasopressor therapy, or cardiac output-guided therapy. Despite the availability of such strategies and the availability of non-invasive cardiac output monitoring, these alternative approaches have not been studied.

Understanding which patient will be fluid responsive is a vital part of rationalising fluid therapy [69]. However, there are multiple different definitions of fluid responsiveness, each dependent on different interventions and different measurements. It would appear that there is little evidence to suggest a consistently different response to FBT based on pre-intervention physiology, as fluid responsiveness is often tautologically and retrospectively defined by participants’ responses to the therapy. A full review of this topic is beyond the scope of this review, though this information is available elsewhere [69,70].

The contribution of FBT to a positive fluid balance remains poorly understood. In a recent observational study, Bihari and colleagues [36] found that a median of 52.4% of fluid balance on the first, 30.8% on the second and 33.2% on the third study day consisted of FBT. In the Fluid and Catheter Treatment Trial [27] and Sepsis Occurrence in Acutely Ill Patients [71] studies, increasing fluid balance was associated with increased risk of acute kidney injury and mortality. In a retrospective study of septic shock patients in a North American university hospital, non-survivors had a significantly greater positive net fluid balance than survivors over the first 24 hours from onset [34]. Our study also shows little or no evidence for any persisting beneficial physiological changes following FBT. These observations suggest the need for RCTs comparing FBT with alternative interventions and well-defined triggers and physiological outcomes.

This review has several strengths. To our knowledge this is the first review of the contemporary literature on FBT in critically ill patients with severe sepsis.

We are the first to explore the contemporary features of a FBT, and the first to produce a summary of the physiological changes associated with FBT in septic, critically ill patients, including data from RCTs, and observational and quasi-experimental studies. Our wide search criteria, use of three separate sources and hand searching references reduced the risk of inclusion bias and makes it unlikely that we missed relevant studies.

Our study also has some limitations. Our assessments of physiological changes are necessarily limited to the measures of central tendency provided in tables and graphs in the studies identified. We have only provided crude median results in an attempt to provide a rough estimate of possible effect. We limited our search to the present evolving decade. It is unlikely that current clinical practice is better reflected by earlier studies. Indeed, in comparing our results with similar, earlier studies, the reported physiological changes are similar [14,71-75]. We did not account for the effect of vasoactive medications beyond noting their administration. It appears obvious that the mixed and differential inotropic/vasopressor/lusitropic/chronotropic effects of different vasoactive medications are likely to have an effect on the physiological changes reported, as would the administration of blood products. Inadequate information was provided in the studies to make such adjustments possible. FBT is normally part of a complex intervention - the resuscitation of the critically ill patient. As well as the initiation and manipulation of vasoactive medications, analyses must contend with the impact of the use of mechanical ventilation, CRRT, and antibiotic administration. These confounders were not reliably reported in the studies identified and could not be evaluated. In addition, the perceived haemodynamic success of an intervention often depends on the trajectory of the patient’s clinical course. Unfortunately no such information was available from the studies reviewed.

## Conclusion

FBT in severe sepsis and septic shock is described in 33 articles in the contemporary literature. Only 17 of these studies report the physiological changes associated with FBT. Evidence regarding the efficacy of FBT compared with alternative interventions is lacking. Crucially, no studies relate the physiological changes after FBT to clinically relevant outcomes. In light of recent studies highlighting the association between FBT and fluid administration in general and harm, there is a clear need for at least obtaining randomised controlled evidence for the physiological effects of FBT over the immediate (0 to 4 hours) post-intervention period in patients with severe sepsis and septic shock.

## Additional file

Additional file 1:
**Electronic Supplement. **Containing: Appendix 1 (Electronic Search Strategies); **Table S1:** Study inclusion criteria, definitions of sepsis and definitions of hyperlactataemia; **Table S2:** Physiological effects grouped by intervention type and comparison; **Table S3:** Physiological effects grouped by speed of FBT delivery; **Table S4:** Physiological effects of FBT grouped by fluid class.

## Abbreviations

CRRT: Continuous renal replacement therapy
CVP: Central venous pressure
FBT: Fluid bolus therapy
IV: Intravenous
RCT: Randomised controlled trial

## References

[1] Holliday MA, Segar WE (1957). The maintenance need for water in parenteral fluid therapy. Pediatrics.

[2] Network SIG (2004). Postoperative management in adults. A practical guide to postoperative care for clinical staff. SIGN Guidelines.

[3] Powell-Tuck J, Gosling P, Lobo DN, Allison SP, Carlson GL, Gore M, Lewington AJ, Pearse RM, Mythen MG: **British consensus guidelines on intravenous fluid therapy for adult surgical patients (GIFTASUP).** [http://www.bapen.org.uk/pdfs/bapen_pubs/giftasup.pdf]

[4] Resuscitation Council UK (2011). Advanced Life Support Manual.

[5] Carey JS, Mohr PA, Brown RS, Shoemaker WC (1969). Cardiovascular function in hemorrhage, trauma and sepsis: determinants of cardiac output and cardiac work. Ann Surg.

[6] Hall JE, Hall JE, Guyton AC (2011). Cardiac output, venous return and their regulation. Guyton and Hall Textbook of Medical Physiology.

[7] Shoemaker WC (1965). Pathophysiologic mechanisms in shock and their therapeutic implications. Am J Surg.

[8] Shoemaker WC, Carey JS, Mohr PA, Brown RS, Monson DO, Yao ST, Kho LK, Stevenson A (1966). Hemodynamic measurements in various types of clinical shock. Analysis of cardiac output and derived calculations in 100 surgical patients. Arch Surg.

[9] Udhoji VN, Weil MH, Sambhi MP, Rosoff L (1963). Hemodynamic studies on clinical shock associated with infection. Am J Med.

[10] Weil MH (1957). Current concepts on the management of shock. Circulation.

[11] Vincent JL, Weil MH (2006). Fluid challenge revisited. Crit Care Med.

[12] Weil MH, Henning RJ (1979). New concepts in the diagnosis and fluid treatment of circulatory shock. Thirteenth annual Becton, Dickinson and Company Oscar Schwidetsky Memorial Lecture. Anesth Analg.

[13] Weil MH, Shubin H, Rosoff L (1965). Fluid repletion in circulatory shock: central venous pressure and other practical guides. JAMA.

[14] Shoemaker WC (1976). Comparison of the relative effectiveness of whole blood transfusions and various types of fluid therapy in resuscitation. Crit Care Med.

[15] Shoemaker WC (1982). Evaluation of colloids, crystalloids, whole blood, and red cell therapy in the critically ill patient. Clin Lab Med.

[16] Shoemaker WC, Hopkins JA, Greenfield S, Chang PC, Umof P, Shabot MM, Spenler CW, State D (1978). Resuscitation algorithm for management of acute emergencies. JACEP.

[17] Muller L, Toumi M, Bousquet PJ, Riu-Poulenc B, Louart G, Candela D, Zoric L, Suehs C, de La Coussaye JE, Molinari N, Lefrant JY, AzuRéa Group (2011). An increase in aortic blood flow after an infusion of 100 ml colloid over 1 minute can predict fluid responsiveness: the mini-fluid challenge study. Anesthesiology.

[18] Dellinger RP, Carlet JM, Masur H, Gerlach H, Calandra T, Cohen J, Gea-Banacloche J, Keh D, Marshall JC, Parker MM, Ramsay G, Zimmerman JL, Vincent JL, Levy MM (2004). Surviving Sepsis Campaign guidelines for management of severe sepsis and septic shock. Intensive Care Med.

[19] Dellinger RP, Levy MM, Carlet JM, Bion J, Parker MM, Jaeschke R, Reinhart K, Angus DC, Brun-Buisson C, Beale R, Calandra T, Dhainaut JF, Gerlach H, Harvey M, Marini JJ, Marshall J, Ranieri M, Ramsay G, Sevransky J, Thompson BT, Townsend S, Vender JS, Zimmerman JL, Vincent JL, International Surviving Sepsis Campaign Guidelines Committee; American Association of Critical-Care Nurses; American College of Chest Physicians; American College of Emergency Physicians; Canadian Critical Care Society; European Society of Clinical Microbiology and Infectious Diseases (2008). Surviving Sepsis Campaign: international guidelines for management of severe sepsis and septic shock: 200. Crit Care Med.

[20] Dellinger RP, Levy MM, Rhodes A, Annane D, Gerlach H, Opal SM, Sevransky JE, Sprung CL, Douglas IS, Jaeschke R, Osborn TM, Nunnally ME, Townsend SR, Reinhart K, Kleinpell RM, Angus DC, Deutschman CS, Machado FR, Rubenfeld GD, Webb SA, Beale RJ, Vincent JL, Moreno R, Surviving Sepsis Campaign Guidelines Committee including the Pediatric Subgroup (2013). Surviving Sepsis Campaign: International guidelines for management of severe sepsis and septic shock: 2012. Crit Care Med.

[21] McLuckie A, Bersten AD (2009). Shock - an overview. Oh’s Intensive Care Manual.

[22] Hilton AK, Bellomo R (2012). A critique of fluid bolus resuscitation in severe sepsis. Crit Care.

[23] Axler O, Tousignant C, Thompson CR, Dalla’va-Santucci J, Drummond A, Phang PT, Russell JA, Walley KR (1997). Small hemodynamic effect of typical rapid volume infusions in critically ill patients. Crit Care Med.

[24] Bouchard J, Soroko SB, Chertow GM, Himmelfarb J, Ikizler TA, Paganini EP, Mehta RL, Program to Improve Care in Acute Renal Disease (PICARD) Study Group (2009). Fluid accumulation, survival and recovery of kidney function in critically ill patients with acute kidney injury. Kidney Int.

[25] Finfer S, Bellomo R, Boyce N, French J, Myburgh J, Norton R, Safe Study Investigators (2004). A comparison of albumin and saline for fluid resuscitation in the intensive care unit. N Engl J Med.

[26] Finfer S, McEvoy S, Bellomo R, McArthur C, Myburgh J, Norton R (2011). Impact of albumin compared to saline on organ function and mortality of patients with severe sepsis. Intensive Care Med.

[27] Grams ME, Estrella MM, Coresh J, Brower RG, Liu KD, National Heart, Lung, and Blood Institute Acute Respiratory Distress Syndrome Network (2011). Fluid balance, diuretic use, and mortality in acute kidney injury. Clin J Am Soc Nephrol.

[28] Heart N, Wiedemann HP, Wheeler AP, Bernard GR, Thompson BT, Hayden D, deBoisblanc B, Connors AF, Hite RD, Harabin AL, Lung, and Blood Institute Acute Respiratory Distress Syndrome (ARDS) Clinical Trials Network (2006). Comparison of two fluid-management strategies in acute lung injury. N Engl J Med.

[29] Berg S, Engman A, Hesselvik JF, Laurent TC (1994). Crystalloid infusion increases plasma hyaluronan. Crit Care Med.

[30] Berg S, Golster M, Lisander B (2002). Albumin extravasation and tissue washout of hyaluronan after plasma volume expansion with crystalloid or hypooncotic colloid solutions. Acta Anaesthesiol Scand.

[31] Steppan J, Hofer S, Funke B, Brenner T, Henrich M, Martin E, Weitz J, Hofmann U, Weigand MA (2011). Sepsis and major abdominal surgery lead to flaking of the endothelial glycocalix. J Surg Res.

[32] Burke-Gaffney A, Evans TW (2012). Lest we forget the endothelial glycocalyx in sepsis. Crit Care.

[33] Woodcock TE, Woodcock TM (2012). Revised Starling equation and the glycocalyx model of transvascular fluid exchange: an improved paradigm for prescribing intravenous fluid therapy. Br J Anaesth.

[34] Hilton AK, Bellomo R (2011). Totem and taboo: fluids in sepsis. Crit Care.

[35] Reade MC, Huang DT, Bell D, Coats TJ, Cross AM, Moran JL, Peake SL, Singer M, Yealy DM, Angus DC, British Association for Emergency Medicine; UK Intensive Care Society; UK Society for Acute Medicine; Australasian Resuscitation in Sepsis Evaluation Investigators; Protocolized Care for Early Septic Shock Investigators (2010). Variability in management of early severe sepsis. Emerg Med J.

[36] Bihari S, Prakash S, Bersten AD (2013). Post resusicitation fluid boluses in severe sepsis or septic shock: prevalence and efficacy (price study). Shock.

[37] Castellanos-Ortega A, Suberviola B, Garcia-Astudillo LA, Holanda MS, Ortiz F, Llorca J, Delgado-Rodriguez M (2010). Impact of the Surviving Sepsis Campaign protocols on hospital length of stay and mortality in septic shock patients: results of a three-year follow-up quasi-experimental study. Crit Care Med.

[38] De Backer D, Biston P, Devriendt J, Madl C, Chochrad D, Aldecoa C, Brasseur A, Defrance P, Gottignies P, Vincent JL, SOAP II Investigators (2010). Comparison of dopamine and norepinephrine in the treatment of shock. N Engl J Med.

[39] Dong ZZ, Fang Q, Zheng X, Shi H (2012). Passive leg raising as an indicator of fluid responsiveness in patients with severe sepsis. World J Emerg Med.

[40] Freitas FGR, Bafi AT, Nascente APM, Assuncao M, Mazza B, Azevedo LCP, Machado FR, Mahajan RP (2013). Predictive value of pulse pressure variation for fluid responsiveness in septic patients using lung-protective ventilation strategies. Br J Anaesth.

[41] Gaieski DF, Mikkelsen ME, Band RA, Pines JM, Massone R, Furia FF, Shofer FS, Goyal M (2010). Impact of time to antibiotics on survival in patients with severe sepsis or septic shock in whom early goal-directed therapy was initiated in the emergency department. Crit Care Med.

[42] Hamzaoui O, Georger JF, Monnet X, Ksouri H, Maizel J, Richard C, Teboul JL (2010). Early administration of norepinephrine increases cardiac preload and cardiac output in septic patients with life-threatening hypotension. Crit Care.

[43] Hanzelka KM, Yeung SC, Chisholm G, Merriman KW, Gaeta S, Malik I, Rice TW (2013). Implementation of modified early-goal directed therapy for sepsis in the emergency center of a comprehensive cancer center. Support Care Cancer.

[44] Jacob ST, Banura P, Baeten JM, Moore CC, Meya D, Nakiyingi L, Burke R, Horton CL, Iga B, Wald A, Reynolds SJ, Mayanja-Kizza H, Scheld WM, Promoting Resource-Limited Interventions for Sepsis Management in Uganda Study Group (2012). The impact of early monitored management on survival in hospitalized adult Ugandan patients with severe sepsis: a prospective intervention study. Crit Care Med.

[45] Khwannimit B, Bhurayanontachai R (2012). Prediction of fluid responsiveness in septic shock patients: comparing stroke volume variation by FloTrac/Vigileo and automated pulse pressure variation. Eur J Anaesthesiol.

[46] Lakhal K, Ehrmann S, Perrotin D, Wolff M, Boulain T (2013). Fluid challenge: tracking changes in cardiac output with blood pressure monitoring (invasive or non-invasive). Intensive Care Med.

[47] Lanspa MJ, Brown SM, Hirshberg EL, Jones JP, Grissom CK (2012). Central venous pressure and shock index predict lack of hemodynamic response to volume expansion in septic shock: a prospective, observational study. J Crit Care.

[48] Machare-Delgado E, Decaro M, Marik PE (2011). Inferior vena cava variation compared to pulse contour analysis as predictors of fluid responsiveness: a prospective cohort study. J Intensive Care Med.

[49] MacRedmond R, Hollohan K, Stenstrom R, Nebre R, Jaswal D, Dodek P (2010). Introduction of a comprehensive management protocol for severe sepsis is associated with sustained improvements in timeliness of care and survival. Qual Saf Health Care.

[50] Mahjoub Y, Benoit-Fallet H, Airapetian N, Lorne E, Levrard M, Seydi AA, Amennouche N, Slama M, Dupont H (2012). Improvement of left ventricular relaxation as assessed by tissue Doppler imaging in fluid-responsive critically ill septic patients. Intensive Care Med.

[51] McIntyre LA, Fergusson DA, Cook DJ, Rowe BH, Bagshaw SM, Easton D, Emond M, Finfer S, Fox-Robichaud A, Gaudert C, Green R, Hebert P, Marshall J, Rankin N, Stiell I, Tinmouth A, Pagliarello J, Turgeon AF, Worster A, Zarychanski R, Canadian Critical Care Trials Group (2012). Fluid resuscitation with 5% albumin versus normal saline in early septic shock: a pilot randomized, controlled trial. J Crit Care.

[52] Monnet X, Anguel N, Naudin B, Jabot J, Richard C, Teboul J (2010). Arterial pressure-based cardiac output in septic patients: different accuracy of pulse contour and uncalibrated pressure waveform devices. Crit Care.

[53] Monnet X, Jabot J, Maizel J, Richard C, Teboul JL (2011). Norepinephrine increases cardiac preload and reduces preload dependency assessed by passive leg raising in septic shock patients. Crit Care Med.

[54] Monnet X, Julien F, Ait-Hamou N, Lequoy M, Gosset C, Jozwiak M, Persichini R, Anguel N, Richard C, Teboul JL (2013). Lactate and venoarterial carbon dioxide difference/arterial-venous oxygen difference ratio, but not central venous oxygen saturation, predict increase in oxygen consumption in fluid responders. Crit Care Med.

[55] Monnet X, Letierce A, Hamzaoui O, Chemla D, Anguel N, Osman D, Richard C, Teboul JL (2011). Arterial pressure allows monitoring the changes in cardiac output induced by volume expansion but not by norepinephrine. Crit Care Med.

[56] O’Neill R, Morales J, Jule M (2012). Early goal-directed therapy (EGDT) for severe sepsis/septic shock: which components of treatment are more difficult to implement in a community-based emergency department?. J Emerg Med.

[57] Ospina-Tascon G, Neves AP, Occhipinti G, Donadello K, Büchele G, Simion D, Chierego ML, Silva TO, Fonseca A, Vincent JL, De Backer D (2010). Effects of fluids on microvascular perfusion in patients with severe sepsis. Intensive Care Med.

[58] Patel GW, Roderman N, Gehring H, Saad J, Bartek W (2010). Assessing the effect of the Surviving Sepsis Campaign treatment guidelines on clinical outcomes in a community hospital. Ann Pharmacother.

[59] Pierrakos C, Velissaris D, Scolletta S, Heenen S, De Backer D, Vincent JL (2012). Can changes in arterial pressure be used to detect changes in cardiac index during fluid challenge in patients with septic shock?. Intensive Care Med.

[60] Pottecher J, Deruddre S, Teboul JL, Georger JF, Laplace C, Benhamou D, Vicaut E, Duranteau J (2010). Both passive leg raising and intravascular volume expansion improve sublingual microcirculatory perfusion in severe sepsis and septic shock patients. Intensive Care Med.

[61] Sanchez M, Jimenez-Lendinez M, Cidoncha M, Asensio MJ, Herrero E, Collado A, Santacruz M (2011). Comparison of fluid compartments and fluid responsiveness in septic and non-septic patients. Anaesth Intensive Care.

[62] Schnell D, Camous L, Guyomarc’h S, Duranteau J, Canet E, Gery P, Dumenil AS, Zeni F, Azoulay E, Darmon M (2013). Renal perfusion assessment by renal Doppler during fluid challenge in sepsis. Crit Care Med.

[63] Sturgess DJ, Pascoe RLS, Scalia G, Venkatesh B (2010). A comparison of transcutaneous Doppler corrected flow time, b-type natriuretic peptide and central venous pressure as predictors of fluid responsiveness in septic shock: a preliminary evaluation. Anaesth Intensive Care.

[64] Trof RJ, Beishuizen A, Cornet AD, Wit RJ, Girbes AR, Groeneveld AB (2012). Volume-limited versus pressure-limited hemodynamic management in septic and nonseptic shock. Crit Care Med.

[65] van Haren FMP, Sleigh J, Boerma EC, La Pine M, Bahr M, Pickkers P, Van Der Hoeven JG (2012). Hypertonic fluid administration in patients with septic shock: a prospective randomized controlled pilot study. Shock.

[66] Wacharasint P, Lertamornpong A, Wattanathum A, Wongsa A (2012). Predicting fluid responsiveness in septic shock patients by using 3 dynamic indices: is it all equally effective?. J Med Assoc Thai.

[67] Yu M, Pei K, Moran S, Edwards KD, Domingo S, Steinemann S, Ghows M, Takiguchi S, Tan A, Lurie F, Takanishi D (2011). A prospective randomized trial using blood volume analysis in addition to pulmonary artery catheter, compared with pulmonary artery catheter alone, to guide shock resuscitation in critically ill surgical patients. Shock.

[68] Zhang Z, Lu B, Ni H, Sheng X, Jin N (2012). Prediction of pulmonary edema by plasma protein levels in patients with sepsis. J Crit Care.

[69] Marik PE, Lemson J (2014). Fluid responsiveness: an evolution of our understanding. Br J Anaesth.

[70] Marik PE, Cavallazzi R (2013). Does the central venous pressure predict fluid responsiveness? An updated meta-analysis and a plea for some common sense. Crit Care Med.

[71] Payen D, de Pont AC, Sakr Y, Spies C, Reinhart K, Vincent JL (2008). Sepsis Occurrence in Acutely Ill Patients Investigators: A positive fluid balance is associated with a worse outcome in patients with acute renal failure. Crit Care.

[72] Calvin JE, Driedger AA, Sibbald WJ (1981). The hemodynamic effect of rapid fluid infusion in critically ill patients. Surgery.

[73] Perner A, Faber T (2006). Stroke volume variation does not predict fluid responsiveness in patients with septic shock on pressure support ventilation. Acta Anaesthesiol Scand.

[74] Sakka SG, Meier-Hellmann A, Reinhart K (2000). Do fluid administration and reduction in norepinephrine dose improve global and splanchnic haemodynamics?. Br J Anaesth.

[75] Shoemaker WC, Schluchter M, Hopkins JA, Appel PL, Schwartz S, Chang PC (1981). Comparison of the relative effectiveness of colloids and crystalloids in emergency resuscitation. Am J Surg.

