# Supplementary material for: Physiological changes after fluid bolus therapy in sepsis: a systematic review of contemporary data
Source: Crit Care. 2014 Dec 27;18(6):696. doi: 10.1186/s13054-014-0696-5 (PMC4331149; doi:10.1186/s13054-014-0696-5)
Supplement: Additional file 1: — Electronic Supplement. Containing: Appendix 1 (Electronic Search Strategies); Table S1: Study inclusion criteria, definitions of sepsis and definitions of hyperlactataemia; Table S2: Physiological effects grouped by intervention type and comparison; Table S3: Physiological effects grouped by speed of FBT delivery; Table S4: Physiological effects of FBT grouped by fluid class. [file 13054_2014_696_MOESM1_ESM.docx]

**Supplemental Electronic Material**

**Appendix 1: Electronic Search Strategies**

**OVID Medline Search Strategy**

1. exp fluid therapy/

2. fluid bolus.tw.

3. fluid therapy.tw.

4. fluid resuscitation.tw.

5. fluid loading.tw.

6. exp oxygen consumption/

7. oxygen delivery.tw.

8. exp cardiac output/

9. cardiac index.tw.

10. optimisation.tw.

11. optimization.tw.

12. goal target.tw.

13. goal directed.tw.

14. goal orientated.tw.

15. goal oriented.tw.

16. 1 or 2 or 3 or 4 or 5 or 6 or 7 or 8 or 9 or 10 or 11 or 12 or 13 or 14 or 15

17. exp sepsis/

18. exp septic shock/

19. (sepsis or septic).tw.

20. 17 or 18 or 19

21. 16 and 20

22. controlled clinical trial.pt.

23. randomized controlled trial.pt.

24. (random$ or placebo$ or single blind$ or double blind$ or triple blind$).ti,ab.

25. exp cohort studies/

26. cohort$.tw.

27. epidemiologic methods/

28. exp case-control studies/

29. (case$ and control$).tw.

30. (random sampl$ or random digit$ or random effect$ or random survey or random regression).ti,ab. not "randomized controlled trial".pt.

31. (comment or editorial or meta-analysis or practice-guideline or review or letter or journal correspondence).pt.

32. (animals not humans).sh.

33. (retraction of publication or retracted publication).pt.

34. 22 or 23 or 24 or 25 or 26 or 27 or 28 or 29

35. 30 or 31 or 32 or 33

36. 21 and 34

37. 36 not 35

38. limit 37 to (english language and yr="2010 - 2013")

**CENTRAL Search Strategy**

ID Search Hits

#1 MeSH descriptor: [Oxygen Consumption] explode all trees

#2 (oxygen delivery):ti,ab,kw

#3 (oxygen consumption):ti,kw,ab

#4 MeSH descriptor: [Stroke Volume] explode all trees

#5 (stroke volume):ti,kw,ab

#6 MeSH descriptor: [Cardiac Volume] explode all trees

#7 MeSH descriptor: [Cardiac Output] explode all trees

#8 (cardiac index or cardiac output):ti,ab,kw

#9 optimisation:ti,ab,kw (Word variations have been searched)

#10 optimization:ti,ab,kw

#11 (goal directed):ti,ab,kw

#12 (goal oriented):ti,ab,kw

#13 (goal orientated):ti,ab,kw

#14 (goal target):ti,ab,kw

#15 exp fluid therapy

#16 (fluid or fluids):ti,ab,kw

#17 #1 or #2 or #3 or #4 or #5 or #6 or #7 or #8 or #9 or #10

or #11 or #12 or #13 or #14 or #15 or #16

#18 exp sepsis

#19 exp septic shock

#20 (sepsis or septic shock or septicemic shock or septicaemic

shock or bacteremic shock or bacteraemic shock):ti,ab,kw

#21 #18 or #19 or #20

#22 #17 and #21 Publication Date from 2010 to 2013, in Trials

**EMBASE Search Strategy**

#1.12 #1.11 AND ('case report'/de OR 'clinical article'/de OR 'clinical trial'/de OR 'clinical trial (topic)'/de OR'cohort analysis'/de OR 'comparative study'/de OR 'controlled clinical trial'/de OR 'controlled study'/de OR'double blind procedure'/de OR 'human'/de OR 'major clinical study'/de OR 'medical record review'/de OR'multicenter study'/de OR 'multicenter study (topic)'/de OR 'observational study'/de OR 'prospective study'/de OR 'randomized controlled trial'/de OR 'randomized controlled trial (topic)'/de OR 'retrospective study'/de OR 'systematic review'/de) AND (2010:py OR 2011:py OR 2012:py OR 2013:py) AND ('article'/it OR'article in press'/it)

#1.11 #1.10 AND [humans]/lim AND [english]/lim AND [embase]/lim AND [2010-2014]/py

#1.10 #1.1 AND #1.6 AND #1.9

#1.9 #1.7 OR #1.8

#1.8 'fluid resuscitation'/exp OR 'fluid resuscitation':ab,ti OR 'fluid bolus':ab,ti OR 'fluid therapy':ab,ti OR 'fluid therapy'/exp OR'intravenous administration'/exp

#1.7 'heart output'/exp OR 'heart output measurement'/exp OR 'heart volume'/exp OR 'heart stroke volume'/exp OR 'cardiac output':ab,ti OR 'cardiac output measurement':ab,ti OR 'cardiac volume':ab,ti OR 'stroke volume':ab,ti OR 'cardiac index':ab,ti

#1.6 #1.4 OR #1.5

#1.5 'longitudinal study'/exp OR 'clinical study'/exp OR 'case control study'/exp OR 'retrospective study'/exp OR 'prospective study'/exp OR 'cohort analysis'/exp

#1.4 #1.2 NOT #1.3

#1.3 'random sampl$':ab,ti OR 'random digit$':ab,ti OR 'random effect$':ab,ti OR 'random survey':ab,ti OR 'random regression':ab,ti

#1.2 'randomized controlled trial'/exp OR 'randomized controlled trial' OR 'controlled clinical trial'/exp OR 'controlled clinical trial'OR 'randomized':ab,ti OR 'placebo':ab,ti OR 'randomly':ab,ti OR 'trial':ab,ti

#1.1 'sepsis'/exp OR 'sepsis' OR 'infection'/exp OR 'infection' OR 'bacteremia'/exp OR 'bacteremia' OR 'septicemia'/exp OR 'septicemia'OR 'sepsis':ab,ti OR 'septic':ab,ti OR 'infection':ab,ti OR 'bacteremia':ab,ti OR 'septicemia':ab,ti OR 'septicaemia':ab,ti OR'bacteraemia':ab,ti

Table S1: Study inclusion criteria, definitions of sepsis and definitions of hyperlactataemia.

| Author | Consensus definition of sepsis? | Volume of pre-inclusion resuscitation. | Haemodynamic criteria for study inclusion. | Definition of hyperlactataemia. |
| --- | --- | --- | --- | --- |
| Aman, et al. | Yes | Not documented. | SBP < 110 mmHg, PCWP < 10 mmHg or CVP < 12 mmHg (PEEP dependent). |  |
| Annane, et al. | No | Not documented. | SBP < 90 or MAP < 60 mmHg; orthostatic hypotension > 20mmHg; clinical evidence of low filling pressure, hyperlactataemia, low CI or poor tissue or organ perfusion. | > 2 mmol/l |
| Arabi, et al. | Yes | Not documented. | As per consensus definitions. |  |
| Bayer, et al. | Yes | Not documented. | As per consensus definitions. |  |
| Bayer, et al. | Yes | "Adequate" resuscitation | SBP ≤ 90 mmHg or MAP ≤ 70 mmHg for > 2 hours or need for vasopressor. | > 2.2 mmol/l |
| Beale, et al | No | "Adequate" resuscitation | SBP < 90mmHg; MAP < 70mmHg for >1 hour; vasoactives; > 40 mmHg drop in SBP. | 1.5 x normal |
| Bihari, et al. | Yes | Not documented. | As per consensus definitions. |  |
| Bouferrache, et al. | Yes | Not documented. | As per consensus definitions. |  |
| Boyd, et al | Yes | 500ml 0.9% saline | Fluid resistant hypotension or requiring low dose noradrenaline. |  |
| Carlsen, et al. | Yes | Not documented. | As per consensus definitions. | > 2 mmol/l |
| Casserly, et al. | No | 30 ml/kg crystalloid | Persistent hypotension | > 4 mmol/l |
| Castellanos-Ortega, et al. | Yes | Not documented. | As per consensus definitions. |  |
| Cronshaw, et al. | Yes | Not documented. | As per consensus definitions. | > 2 mmol/l |
| De Backer, et al. | ND | 500 ml colloid or 1000 ml crystalloid | MAP < 70 mmHg; SBP < 100 mmHg, altered mental state; mottled skin; oliguria > 1 hour, hyperlactataemia. | > 2 mmol/l |
| Dong, et al. | Yes | Not documented. | SBP < 90mmHg or >40mmHg drop or need for vasopressors, oliguria > 1 hour; mottled skin; HR > 100 bpm |  |
| Dubin, et al. | Yes | 20 ml/kg crystalloid | MAP < 65 mmHg despite 20ml/kg crystalloid | > 4 mmol/l |
| Finfer, et al. | Yes | Not documented. | SOFA Organ Dysfunction: Renal > 2; Respiratory, Haematological, Hepatic > 1; Cardiovascular 1, 3, 4. |  |
| Freitas, et al. | Yes | Not documented. | As per consensus definitions. |  |
| Gaieski, et al. | Yes | 20-30 ml/kg intravenous fluid | SBP < 90 mmHg. | > 4 mmol/l |
| Guidet, et al. | Yes | Not documented. | As per consensus definitions. |  |
| Gurnani, et al. | Yes | "Adequate" resuscitation | SBP < 90 mmHg or MAP < 65 mmHg. |  |
| Hamzaoui, et al. | Yes | Not documented. | MAP < 65 mmHg |  |
| Hanzelka, et al. | Yes | 20 ml/kg intravenous fluid. | SBP < 90 mmHg or MAP < 65 mmHg | > 4 mmol/l |
| Hartemink, et al. | No | Not documented. | PAOP < 13mmHg or CVP < 12mmHg (PEEP dependent) with SBP < 110mmHg without inotrope use in the absence of overhydration. |  |
| Huh, et al. | Yes | Not documented. | As per consensus definitions. |  |
| Jacob, et al. | Yes | Not documented. | SBP < 100 mmHg or hyperlactataemia. | > 2.5 mmol/l |
| Jones, et al. | Yes | 20 ml/kg intravenous fluid. | SBP < 90 mmHg | > 4 mmol/l |
| Khwannimit, et al | Yes | Not documented. | MAP < 65 mmHg, oliguria, tachycardia, skin mottling. |  |
| Lakhal, et al. | No | Not documented. | One or more of SBP < 90 mmHg, MAP < 65 mmHg , requiring vasoactive medication, oliguria, skin mottlin, hyperlactataemia | > 2.5 mmol/l |
| Lanspa, et al. | Yes | 20 ml/kg intravenous fluid. | SBP < 90 mmHg |  |
| Machare-Delgado, et al. | No | Not documented. | Low blood pressure or evidence of organ dysfunction |  |
| MacRedmond, et al. | Yes | 25 ml/kg intravenous fluid. | SBP < 90 mmHg | > 4 mmol/l |
| Mahjoub, et al. | ND | Not documented. | SBP < 90 mmHg and/or need for vasoactive drugsand/or persistent lactic acidosis. | Lactic acidosis |
| McIntyre, et al. | Yes | 1000 ml crystalloid. | MAP < 65 mmHg, SBP < 90 mmHg or 40 mmHg decrease in SBP or requiring vasopressors within 8 hours of first hypotensive episode. |  |
| Mikkelsen, et al. | Yes | 1500 ml intravenous fluid. | SBP < 90 mmHg. | > 4 mmol/l |
| Monnet, et al. | No | Not documented. | SBP < 90 mmHg, SBP drop > 50mmHg if HT, and one or more of HR > 100, skin mottling or oliguria |  |
| Monnet, et al. | No | Not documented. | SBP < 90 mmHg, SBP drop > 50mmHg if HT, and one or more of HR > 100, skin mottling or oliguria |  |
| Monnet, et al. | No | Not documented. | SBP < 90 mmHg, SBP drop > 50mmHg if HT, and one or more of HR > 100, skin mottling or oliguria |  |
| Monnet, et al. | No | Not documented. | SBP < 90 mmHg, SBP drop > 50mmHg if HT, and one or more of HR > 100, skin mottling or oliguria |  |
| Muller, et al | Yes | Not documented. | As per consensus definitions. |  |
| Napoli, et al. | Yes | 2000 ml intravenous fluid. | SBP < 90 mmHg or MAP < 65 mmHg. | > 4 mmol/l |
| Nguyen, et al | Yes | 1000 ml crystalloid. | SBP < 90 mm Hg, two or more organ system dysfunctions. | > 4 mmol/l |
| O'Neill, et al. | Yes | 20 ml/kg intravenous fluid. | MAP < 65 mmHg | > 4 mmol/l |
| Ospina-Tascon, et al. | Yes | Not documented. | MAP < 65 mmHg | > 2 mmol/l |
| Patel, et al. | Yes | Not documented. | SBP < 90 mmHg or MAP < 65 mmHg and evidence of organ dysfunction |  |
| Perner, et al. | Yes | Not documented. | As per consensus definitions. | > 2 mmol/l |
| Phua, et al. | Yes | Not documented. | SBP < 90 mmHg; MAP < 65 mmHg or SBP drop > 40 mmHg | > 2 mmol/l |
| Pierrakos, et al. | Yes | Not documented. | As per consensus definitions. |  |
| Pottecher, et al. | Yes | Not documented. | MAP < 65 mmHg, skin mottling or oliguria |  |
| Puskarich, et al. | Yes | 20 ml/kg intravenous fluid. | SBP < 90 mmHg | > 4 mmol/l |
| Puskarich, et al. | Yes | 20 ml/kg intravenous fluid. | SBP < 90 mmHg | > 4 mmol/l |
| Puskarich, et al. | Yes | 20 ml/kg intravenous fluid. | SBP < 90 mmHg | > 4 mmol/l |
| Rincon, et al. | Yes | Not documented. | SBP < 90 mmHg; MAP < 65 mmHg | > 4 mmol/l |
| Sanchez, et al. | Yes | "Adequate" resuscitation | Hypotension with perfusion abnormalities. | Elevated. |
| Schnell, et al. | Yes | Not documented. | As per consensus definitions. |  |
| Schortgen, et al. | Yes | Not documented. | 2 or more of SBP < 90 mmHg; SBP drop > 40mmHg, need for vasoactives, HR > 90 bpm, skin mottling, RR > 25/min, mechanical ventilation, acute confusion, UO < 30ml/hr. | > 2 mmol/l |
| Seymour, et al. | Yes | 1500 ml intravenous fluid. | As per consensus definitions. | > 2 mmol/l |
| Sturgess, et al. | Yes | Not documented. | As per consensus definitions. |  |
| Trof, et al. | Yes | Not documented. | SBP < 110 mmHg; CVP < 12 mmHg (PEEP dependent) in the absence of overt bleeding. |  |
| Trof, et al. | Yes | "Adequate" resuscitation | MAP < 65 mmHg and/or the need for vasopressor medication. |  |
| van Haren, et al. | Yes | Not documented. | As per consensus definitions. |  |
| Wacharasint, et al. | Yes | Not documented. | SBP < 90 mmHg or requirement for vasopressors. |  |
| Yu, et al. | Yes | 30 ml/kg intravenous fluid. | SBP > 90mmHg or > 4 0mmHg drop in BP. | Elevated. |
| Zhang, et al. | Yes | Not documented. | As per consensus definitions. |  |

Table S2: Physiological effects grouped by intervention type and comparison

| **Author** | **Intervention/Fluid Given** | **Group** | **Time from completion of fluid administration until physiological measurement** | **Measure of Central Tendency** | **Change in Cardiac Output Estimation** | **Change in heart rate (bpm )** | **Change in Systemic Pressure (mmHg))** | **Change in central venous pressuree (mmHg)** | **Change in venous oxygen saturation (%)** | **Change in blood lactate concentration (mmol/l)** | **Change in urine output (ml/h)** | **Change in haemoglobin concentration (g/L)** |
| --- | --- | --- | --- | --- | --- | --- | --- | --- | --- | --- | --- | --- |
| **Studies comparing intervention types or other groups** | | | | | | | | | | | | |
| Monnet [55] | 500ml of 0.9% saline over 20 minutes | Responders: >15% CI increase | 0 minutes. | Mean | +800 ml/min/m2 | -2 | +11 |  |  |  |  |  |
|  | 500ml of 0.9% saline over 20 minutes | Non-responders: < 15% increase in CI | 0 minutes. | Mean | +200 ml/min/m2 | -2 | +4 |  |  |  |  |  |
|  | Initiation of noradrenaline at 0.24 (0.13 - 0.48) mcg/kg/min or increase from 0.48 (0.24 - 0.71) to 0.62 (0.43 - 1.07) mcg/kg/min |  | 0 minutes | Mean | +400 ml/min/m2 | -1 | +21 |  |  |  |  |  |
| van Haren [65] | 250ml of 6% HES in 7.2% saline over 15 minutes. | Hypertonic bolus | 30 minutes | Mean | +300 ml/ml/m2 | -11 | +4 | +2 |  | -0.2 |  | -8 |
|  | 500ml of 6% HES in 0.9% saline over 15 minutes. | Isotonic bolus | 30 minutes | Mean | - 400 ml/min/m2 | -1 | +5 | +4 |  | -0.1 |  | -9 |
|  | 250ml of 6% HES in 7.2% saline over 15 minutes. | Hypertonic bolus | 60 minutes | Mean | +400 ml/min/m2 | -11 | +6 | +1 |  | -0.3 |  | -9 |
|  | 500ml of 6% HES in 0.9% saline over 15 minutes. | Isotonic bolus | 60 minutes | Mean | - 300 ml/min/m2 | -1 | +3 | +3 |  | -0.1 |  | -12 |
| Ospina-Tascon [57] | 400ml of 4% albumin or 1000ml of CSL over 30 minutes | Patients with early sepsis | 60 minutes | Median | +300 ml/min/m2 | +2 | +2 | +3 | +2% | -0.2 |  |  |
|  | 400ml of 4% albumin or 1000ml of CSL over 30 minutes | Patients with late sepsis | 60 minutes | Median | +300 ml/min/m2 | -9 | +7 | +1 | +1% | +0.1 |  |  |
| van Haren [65] | 250ml of 6% HES in 7.2% saline over 15 minutes. | Hypertonic bolus | 120 minutes | Mean | +300 ml/ml/m2 | -7 | +7 | +2 |  | +0 | +13 | -6 |
|  | 500ml of 6% HES in 0.9% saline over 15 minutes. | Isotonic bolus | 120 minutes | Mean | - 300 ml/min/m2 | +0 | +1 | +2 |  | -0.3 | -30 | -9 |
|  | 250ml of 6% HES in 7.2% saline over 15 minutes. | Hypertonic bolus | 180 minutes | Mean | +100 ml/min/m2 | -3 | +6 | +3 |  | -0.3 |  | -9 |
|  | 500ml of 6% HES in 0.9% saline over 15 minutes. | Isotonic bolus | 180 minutes | Mean | + 0 ml/min/m2 | +3 | +5 | +3 |  | -0.2 |  | -6 |
|  | 250ml of 6% HES in 7.2% saline over 15 minutes. | Hypertonic bolus | 240 minutes | Mean | +100 ml/min/m2 | +1 | +3 | +3 |  | -0.3 | -3 | -8 |
|  | 500ml of 6% HES in 0.9% saline over 15 minutes. | Isotonic bolus | 240 minutes | Mean | -200 ml/min/m2 | +3 | +0 | +3 |  | -0.2 | -40 | -4 |
|  | | | | | | | | | | | | |
|  | | | | | | | | | | | | |
|  | | | | | | | | | | | | |
|  | | | | | | | | | | | | |
| **Author** | **Intervention/Fluid Given** | **Group** | **Time from completion of fluid administration until physiological measurement** | **Measure of Central Tendency** | **Change in Cardiac Output Estimation** | **Change in heart rate (bpm )** | **Change in Systemic Pressure (mmHg))** | **Change in central venous pressuree (mmHg)** | **Change in venous oxygen saturation (%)** | **Change in blood lactate concentration (mmol/l)** | **Change in urine output (ml/h)** | **Change in haemoglobin concentration (g/L)** |
| **Studies comparing haemodynamic responders with non-responders** | | | | | | | | | | | | |
| *Responders* | | | | | | | | | | | | |
| Dong [39] | 500ml of 6% HES over 30 minutes | Responders: > 15% SVI increase | 0 minutes. | Mean | +600 ml/min/m2 | -1.5 | +15.2 | +3.2 |  |  |  |  |
| Khwannimit [45] | 500ml of 6% HES over 30 minutes | Responders: >15% SVI increase | 0 minutes. | Mean | +1300 ml/min/m2 | -3.3 | +9.5 | +3.4 |  |  |  |  |
| Lakhal [46] | 500ml of 4% gelatin over 30 minutes | Responders: >15% SVI increase | 0 minutes. | Mean | +900 ml/min/m2 | -6 | +14 | +3 |  |  |  |  |
| Machare-Delgado [48] | 500ml of 0.9% saline over 10 minutes | Responders: >10% SVI increase | 0 minutes | Mean | +3.99 ml/m2/beat |  |  |  |  |  |  |  |
| Mahjoub [50] | 500ml of 0.9% saline over 20 minutes | Responders: >10% SV increase | 0 minutes. | Mean | +1000 ml/min | -4 | +7 | +2.6 |  |  |  |  |
| Monnet [55] | 500ml of 0.9% saline over 20 minutes | Responders: >15% CI increase | 0 minutes. | Mean | +800 ml/min/m2 | -2 | +11 |  |  |  |  |  |
| Monnet [54] | 500ml of 0.9% saline over 30 minutes | Responders: >15% VO2 increase | 0 minutes. | Mean | +1000ml/min/m2 | -2 | +7 |  | +1% | -1.9 |  | -7 |
| Schnell [62] | 500ml of 0.9% saline over 15-30 minutes | Responders: > 10% increase in aortic blood flow. | 0 minutes. | Median | +20 ml/beat | -10 | +7 |  |  |  |  |  |
| Freitas [40] | 7ml/kg, max 500ml, of 6% HES over 30 minutes | Responders: > 15% CO increase | 30 minutes | Mean | +2100 ml/min | -2 | +11 | +3 | +8% | -0.11 |  |  |
| Pierrakos [59] | 500ml of 6% HES or 1000ml of CSL over 30 minutes | Responders: > 10% increase in CI. | 30 minutes | Mean | +600 ml/min/m2 | -4 | +8 | +3 | +3% |  |  |  |
| *Non-responders* | | | | | | | | | | | | |
| Dong [39] | 500ml of 6% HES over 30 minutes | Non-responders: < 15% SVI increase | 0 minutes. | Mean | +300 ml/min/m2 | -1.2 | +4.8 | +2.3 |  |  |  |  |
| Khwannimit [45] | 500ml of 6% HES over 30 minutes | Non-responders: <15% SVI increase | 0 minutes. | Mean | +200 ml/min/m2 | -0.9 | +3.9 | +5.2 |  |  |  |  |
| Lakhal [46] | 500ml of 4% gelatin over 30 minutes | Non-responders: <15% SVI increase | 0 minutes. | Mean | +0 ml/min/m2 | -3 | +7 | +4.5 |  |  |  |  |
| Machare-Delgado [48] | 500ml of 0.9% saline over 10 minutes | Non-responders: >10% SVI increase | 0 minutes. | Mean | +0.57 ml/m2/beat |  |  |  |  |  |  |  |
| Mahjoub [50] | 500ml of 0.9% saline over 20 minutes | Non-responders: >10% SV increase | 0 minutes. | Mean | +300 ml/min | -3 | +1 | +2.9 |  |  |  |  |
| Monnet [55] | 500ml of 0.9% saline over 20 minutes | Non-responders: < 15% increase in CI | 0 minutes. | Mean | +200 ml/min/m2 | -2 | +4 |  |  |  |  |  |
| Monnet [54] | 500ml of 0.9% saline over 30 minutes | Non-responders: < 15% increase in VO2 | 0 minutes. | Mean | +1000 ml/min/m2 | +0 | +13 |  | +7% | -0.3 |  | -6 |
| Schnell [62] | 500ml of 0.9% saline over 15-30 minutes | Non-responders: < 10% increase in aortic blood flow. | 0 minutes. | Median | +8 ml/beat | -1 | +6 |  |  |  |  |  |
| Freitas [40] | 7ml/kg, max 500ml, of 6% HES over 30 minutes | Non-responders: < 15% increase in CO | 30 minutes | Mean | +200 ml/min | +0 | +8 | +5 | -3.5% | -0.22 |  |  |
| Pierrakos [59] | 500ml of 6% HES or 1000ml of CSL over 30 minutes | Non-responders: < 10% increase in CI. | 30 minutes | Mean | +0 ml/min/m2 | -4 | +3 | +2 | +0% |  |  |  |
| **Author** | **Intervention/Fluid Given** | **Group** | **Time from completion of fluid administration until physiological measurement** | **Measure of Central Tendency** | **Change in Cardiac Output Estimation** | **Change in heart rate (bpm )** | **Change in Systemic Pressure (mmHg))** | **Change in central venous pressure (mmHg)** | **Change in venous oxygen saturation (%)** | **Change in blood lactate concentration (mmol/l)** | **Change in urine output (ml/h)** | **Change in haemoglobin concentration (g/L)** |
| **Studies examining a single group** | | | | | | | | | | | | |
| Monnet [53] | 500ml of 0.9% saline over 10 minutes | All patients. | 0 minutes. | Mean | +800 ml/min/m2 | -7 | +8 | +5 |  |  |  |  |
| Sturgess [63] | 250ml of 4% Albumin over 15 minutes | All patients | 0 minutes. | Mean | + 7.5% ml/beat |  |  |  |  |  |  |  |
| Pottecher [60] | Up to 500ml of 6% HES or 0.9% saline over 30 minutes | All patients | 30 minutes | Mean | +1400 ml/min | -2 | +7 |  |  |  |  |  |
| Wacharasint [66] | 500ml of 6% HES over 30 minutes | All patients | 30 minutes | Mean | +470 ml/min/m2 | +0.3 | +9.2 | +5.25 |  |  |  |  |
| Bihari [36] | 500 - 750ml of 4% albumin, blood, 20% albumin FFP, 0.9% saline, 4% gelatin or platelts administered over less than 30 minutes. | All patients. | 60 minutes | Median |  | +0 | +2 | +2 | +0.4% | -0.2 | No change | -6 |
| * systolic blood pressure; right atrial pressure; § : mixed venous oxygen saturation; SVC: superior vena cava; HES: hydroxyethyl starch; CSL: compound sodium lactate; SV: stroke volume; SVI strok volume index; CO: cardiac output; CI: cardiac index; VO2: oxygen delivery; | | | | | | | | | | | | |

Table S3: Physiological effects grouped by speed of FBT delivery

| **FIRST AUTHOR** | **Fluid Given** | **Group** | **Time from completion of fluid administration until physiological measurement** | **Measure of Central Tendency** | **Change in Cardiac Output Estimation** | **Change in heart rate (bpm )** | **Change in Mean Arterial Pressure (mmHg))** | **Change in central venous pressure (mmHg)** | **Change in venous oxygen saturation (%)** | **Change in blood lactate concentration (mmol/l)** | **Change in urine output** | **Change in haemoglobin concentration (g/L)** |
| --- | --- | --- | --- | --- | --- | --- | --- | --- | --- | --- | --- | --- |
| **Fluid bolus administered over 10 minutes** | | | | | | | | | | | | |
| Machare-Delgado [48] | 500ml of 0.9% saline over 10 minutes | Responders: >10% SVI increase | 0 minutes | Mean | +3.99 ml/m2/beat |  |  |  |  |  |  |  |
| Machare-Delgado [48] | 500ml of 0.9% saline over 10 minutes | Non-responders: >10% SVI increase | 0 minutes. | Mean | +0.57 ml/m2/beat |  |  |  |  |  |  |  |
| Monnet [53] | 500ml of 0.9% saline over 10 minutes | All patients. | 0 minutes. | Mean | +800 ml/min/m2 | -7 | 8 | 5 |  |  |  |  |
| **Fluid bolus administered over 15 minutes** | | | | | | | | | | | | |
| Sturgess [63] | 250ml of 4% Albumin over 15 minutes | All patients | 0 minutes. | Mean | + 7.5% ml/beat |  |  |  |  |  |  |  |
| van Haren [65] | 250ml of 6% HES in 7.2% saline over 15 minutes. | Hypertonic bolus | 30 minutes | Mean | +300 ml/min/m2 | -11 | 4 | 2 |  | -0.2 |  | -8 |
| van Haren [65] | 500ml of 6% HES in 0.9% saline over 15 minutes. | Isotonic bolus | 30 minutes | Mean | -400 ml/min/m3 | -1 | 5 | 4 |  | -0.1 |  | -9 |
| van Haren [65] | 250ml of 6% HES in 7.2% saline over 15 minutes. | Hypertonic bolus | 60 minutes | Mean | +400 ml/min/m3 | -11 | 6 | 1 |  | -0.3 |  | -9 |
| van Haren [65] | 500ml of 6% HES in 0.9% saline over 15 minutes. | Isotonic bolus | 60 minutes | Mean | -300 ml/min/m4 | -1 | 3 | 3 |  | -0.1 |  | -12 |
| van Haren [65] | 250ml of 6% HES in 7.2% saline over 15 minutes. | Hypertonic bolus | 120 minutes | Mean | +300 ml/ml/m2 | -7 | 7 | 2 |  | 0 | 13 | -6 |
| van Haren [65] | 500ml of 6% HES in 0.9% saline over 15 minutes. | Isotonic bolus | 120 minutes | Mean | - 300 ml/min/m2 | 0 | 1 | 2 |  | -0.3 | -30 | -9 |
| van Haren [65] | 250ml of 6% HES in 7.2% saline over 15 minutes. | Hypertonic bolus | 180 minutes | Mean | +100 ml/min/m2 | -3 | 6 | 3 |  | -0.3 |  | -9 |
| van Haren [65] | 500ml of 6% HES in 0.9% saline over 15 minutes. | Isotonic bolus | 180 minutes | Mean | + 0 ml/min/m2 | 3 | 5 | 3 |  | -0.2 |  | -6 |
| van Haren [65] | 250ml of 6% HES in 7.2% saline over 15 minutes. | Hypertonic bolus | 240 minutes | Mean | +100 ml/min/m2 | 1 | 3 | 3 |  | -0.3 | -3 | -8 |
| van Haren [65] | 500ml of 6% HES in 0.9% saline over 15 minutes. | Isotonic bolus | 240 minutes | Mean | -200 ml/min/m2 | 3 | 0 | 3 |  | -0.2 | -40 | -4 |
| **Fluid bolus administered over 20 minutes** | | | | | | | | | | | | |
| Monnet [55] | 500ml of 0.9% saline over 20 minutes | Responders: >15% CI increase | 0 minutes. | Mean | +800 ml/min/m2 | -2 | 11 |  |  |  |  |  |
| Monnet [55] | 500ml of 0.9% saline over 20 minutes | Non-responders: < 15% increase in CI | 0 minutes. | Mean | +200 ml/min/m2 | -2 | 4 |  |  |  |  |  |
| Mahjoub [50] | 500ml of 0.9% saline over 20 minutes | Responders: >10% SV increase | 0 minutes. | Mean | +1000 ml/min | -4 | 7 | 2.6 |  |  |  |  |
| Mahjoub [50] | 500ml of 0.9% saline over 20 minutes | Non-responders: >10% SV increase | 0 minutes. | Mean | +300 ml/min | -3 | 1 | 2.9 |  |  |  |  |
| **FIRST AUTHOR** | **Fluid Given** | **Group** | **Time from completion of fluid administration until physiological measurement** | **Measure of Central Tendency** | **Change in Cardiac Output Estimation** | **Change in heart rate (bpm )** | **Change in Mean Arterial Pressure (mmHg))** | **Change in central venous pressure (mmHg)** | **Change in venous oxygen saturation (%)** | **Change in blood lactate concentration (mmol/l)** | **Change in urine output** | **Change in haemoglobin concentration (g/L)** |
| **Fluid bolus administered over 30 minutes** | | | | | | | | | | | | |
| Schnell [62] | 500ml of 0.9% saline over 15-30 minutes | Responders: > 10% increase in aortic blood flow. | 0 minutes. | Median | +20 ml/beat | -10 | 7 |  |  |  |  |  |
| Schnell [62] | 500ml of 0.9% saline over 15-30 minutes | Non-responders: < 10% increase in aortic blood flow. | 0 minutes. | Median | +8 ml/beat | -1 | 6 |  |  |  |  |  |
| Dong [39] | 500ml of 6% HES over 30 minutes | Responders: > 15% SVI increase | 0 minutes. | Mean | +600 ml/min/m2 | -1.5 | 15.2 | 3.2 |  |  |  |  |
| Dong [39] | 500ml of 6% HES over 30 minutes | Non-responders: < 15% SVI increase | 0 minutes. | Mean | +300 ml/min/m2 | -1.2 | 4.8 | 2.3 |  |  |  |  |
| Khwannimit [45] | 500ml of 6% HES over 30 minutes | Responders: >15% SVI increase | 0 minutes. | Mean | +1300 ml/min/m2 | -3.3 | 9.5 | 3.4 |  |  |  |  |
| Khwannimit [45] | 500ml of 6% HES over 30 minutes | Non-responders: <15% SVI increase | 0 minutes. | Mean | +200 ml/min/m2 | -0.9 | 3.9 | 5.2 |  |  |  |  |
| Lakhal [46] | 500ml of 4% gelatin over 30 minutes | Responders: >15% SVI increase | 0 minutes. | Mean | +900 ml/min/m2 | -6 | 14 | 3 |  |  |  |  |
| Lakhal [46] | 500ml of 4% gelatin over 30 minutes | Non-responders: <15% SVI increase | 0 minutes. | Mean | +0 ml/min/m2 | -3 | 7 | 4.5 |  |  |  |  |
| Monnet [54] | 500ml of 0.9% saline over 30 minutes | Responders: >15% VO2 increase | 0 minutes. | Mean | +1000ml/min/m2 | -2 | 7 |  | 1% | -1.9 |  | -7 |
| Monnet [54] | 500ml of 0.9% saline over 30 minutes | Non-responders: < 15% increase in VO2 | 0 minutes. | Mean | +1000 ml/min/m2 | 0 | 13 |  | 7% | -0.3 |  | -6 |
| Freitas [40] | 7ml/kg, max 500ml, of 6% HES over 30 minutes | Responders: > 15% CO increase | 30 minutes | Mean | +2100 ml/min | -2 | 11 | 3 | 8% | -0.1 |  |  |
| Freitas [40] | 7ml/kg, max 500ml, of 6% HES over 30 minutes | Non-responders: < 15% increase in CO | 30 minutes | Mean | +200 ml/min | 0 | 8 | 5 | -3.50% | -0.2 |  |  |
| Pierrakos [59] | 500ml of 6% HES or 1000ml of CSL over 30 minutes | Responders: > 10% increase in CI. | 30 minutes | Mean | +600 ml/min/m2 | -4 | 8 | 3 | 3% |  |  |  |
| Pierrakos [59] | 500ml of 6% HES or 1000ml of CSL over 30 minutes | Non-responders: < 10% increase in CI. | 30 minutes | Mean | +0 ml/min/m2 | -4 | 3 | 2 | 0% |  |  |  |
| Pottecher [60] | Up to 500ml of 6% HES or 0.9% saline over 30 minutes | All patients | 30 minutes | Mean | +1400 ml/min | -2 | 7 |  |  |  |  |  |
| Wacharasint [66] | 500ml of 6% HES over 30 minutes | All patients | 30 minutes | Mean | +470 ml/min/m2 | 0.3 | 9.2 | 5.25 |  |  |  |  |
| Bihari [36] | 500 - 750ml of 4% albumin, blood, 20% albumin FFP, 0.9% saline, 4% gelatin or platelts administered over less than 30 minutes. | All patients. | 60 minutes | Median |  | 0 | 2 | 2 | 0.40% | -0.2 | No change | -6 |
| Ospina-Tascon [57] | 400ml of 4% albumin or 1000ml of CSL over 30 minutes | Patients with early sepsis | 60 minutes | Median | +300 ml/min/m2 | 2 | 2 | 3 | 2% | -0.2 |  |  |
| Ospina-Tascon [57] | 400ml of 4% albumin or 1000ml of CSL over 30 minutes | Patients with late sepsis | 60 minutes | Median | +300 ml/min/m2 | -9 | 7 | 1 | 1% | 0.1 |  |  |
| SVC: superior vena cava; HES: hydroxyethyl starch; CSL: compound sodium lactate; SV: stroke volume; SVI stroke volume index; CO: cardiac output; CI: cardiac index; VO2: oxygen delivery; | | | | | | | | | | | | |

Table S4: Physiological effects of FBT grouped by fluid class

| **FIRST AUTHOR** | **Fluid Given** | **Group** | **Time from completion of fluid administration until physiological measurement** | **Measure of Central Tendency** | **Change in Cardiac Output Estimation** | **Change in heart rate (bpm )** | **Change in Mean Arterial Pressure (mmHg))** | **Change in central venous pressure (mmHg)** | **Change in venous oxygen saturation (%)** | **Change in blood lactate concentration (mmol/l)** | **Change in urine output** | **Change in haemoglobin concentration (g/L)** |
| --- | --- | --- | --- | --- | --- | --- | --- | --- | --- | --- | --- | --- |
| **Crystalloid fluid boluses** | | | | | | | | | | | | |
| Machare-Delgado [48] | 500ml of 0.9% saline over 10 minutes | Responders: >10% SVI increase | 0 minutes | Mean | +3.99 ml/m2/beat |  |  |  |  |  |  |  |
| Machare-Delgado [48] | 500ml of 0.9% saline over 10 minutes | Non-responders: >10% SVI increase | 0 minutes. | Mean | +0.57 ml/m2/beat |  |  |  |  |  |  |  |
| Monnet [53] | 500ml of 0.9% saline over 10 minutes | All patients. | 0 minutes. | Mean | +800 ml/min/m2 | -7 | 8 | 5 |  |  |  |  |
| Monnet [55] | 500ml of 0.9% saline over 20 minutes | Responders: >15% CI increase | 0 minutes. | Mean | +800 ml/min/m2 | -2 | 11 |  |  |  |  |  |
| Monnet [55] | 500ml of 0.9% saline over 20 minutes | Non-responders: < 15% increase in CI | 0 minutes. | Mean | +200 ml/min/m2 | -2 | 4 |  |  |  |  |  |
| Mahjoub [50] | 500ml of 0.9% saline over 20 minutes | Responders: >10% SV increase | 0 minutes. | Mean | +1000 ml/min | -4 | 7 | 2.6 |  |  |  |  |
| Mahjoub [50] | 500ml of 0.9% saline over 20 minutes | Non-responders: >10% SV increase | 0 minutes. | Mean | +300 ml/min | -3 | 1 | 2.9 |  |  |  |  |
| Schnell [62] | 500ml of 0.9% saline over 15-30 minutes | Responders: > 10% increase in aortic blood flow. | 0 minutes. | Median | +20 ml/beat | -10 | 7 |  |  |  |  |  |
| Schnell [62] | 500ml of 0.9% saline over 15-30 minutes | Non-responders: < 10% increase in aortic blood flow. | 0 minutes. | Median | +8 ml/beat | -1 | 6 |  |  |  |  |  |
| Monnet [54] | 500ml of 0.9% saline over 30 minutes | Responders: >15% VO2 increase | 0 minutes. | Mean | +1000ml/min/m2 | -2 | 7 |  | 1% | -1.9 |  | -7 |
| Monnet [54] | 500ml of 0.9% saline over 30 minutes | Non-responders: < 15% increase in VO2 | 0 minutes. | Mean | +1000 ml/min/m2 | 0 | 13 |  | 7% | -0.3 |  | -6 |
| **Colloid fluid boluses** | | | | | | | | | | | | |
| Sturgess [63] | 250ml of 4% Albumin over 15 minutes | All patients | 0 minutes. | Mean | + 7.5% ml/beat |  |  |  |  |  |  |  |
| van Haren [65] | 250ml of 6% HES in 7.2% saline over 15 minutes. | Hypertonic bolus | 30 minutes | Mean | +300 ml/min/m2 | -11 | 4 | 2 |  | -0.2 |  | -8 |
| van Haren [65] | 500ml of 6% HES in 0.9% saline over 15 minutes. | Isotonic bolus | 30 minutes | Mean | -400 ml/min/m3 | -1 | 5 | 4 |  | -0.1 |  | -9 |
| van Haren [65] | 250ml of 6% HES in 7.2% saline over 15 minutes. | Hypertonic bolus | 60 minutes | Mean | +400 ml/min/m3 | -11 | 6 | 1 |  | -0.3 |  | -9 |
| **FIRST AUTHOR** | **Fluid Given** | **Group** | **Time from completion of fluid administration until physiological measurement** | **Measure of Central Tendency** | **Change in Cardiac Output Estimation** | **Change in heart rate (bpm )** | **Change in Mean Arterial Pressure (mmHg))** | **Change in central venous pressure (mmHg)** | **Change in venous oxygen saturation (%)** | **Change in blood lactate concentration (mmol/l)** | **Change in urine output** | **Change in haemoglobin concentration (g/L)** |
| **Colloid fluid boluses (contd)** | | | | | | | | | | | | |
| van Haren [65] | 500ml of 6% HES in 0.9% saline over 15 minutes. | Isotonic bolus | 60 minutes | Mean | -300 ml/min/m4 | -1 | 3 | 3 |  | -0.1 |  | -12 |
| van Haren [65] | 250ml of 6% HES in 7.2% saline over 15 minutes. | Hypertonic bolus | 120 minutes | Mean | +300 ml/ml/m2 | -7 | 7 | 2 |  | 0 | 13 | -6 |
| van Haren [65] | 500ml of 6% HES in 0.9% saline over 15 minutes. | Isotonic bolus | 120 minutes | Mean | - 300 ml/min/m2 | 0 | 1 | 2 |  | -0.3 | -30 | -9 |
| van Haren [65] | 250ml of 6% HES in 7.2% saline over 15 minutes. | Hypertonic bolus | 180 minutes | Mean | +100 ml/min/m2 | -3 | 6 | 3 |  | -0.3 |  | -9 |
| van Haren [65] | 500ml of 6% HES in 0.9% saline over 15 minutes. | Isotonic bolus | 180 minutes | Mean | + 0 ml/min/m2 | 3 | 5 | 3 |  | -0.2 |  | -6 |
| van Haren [65] | 250ml of 6% HES in 7.2% saline over 15 minutes. | Hypertonic bolus | 240 minutes | Mean | +100 ml/min/m2 | 1 | 3 | 3 |  | -0.3 | -3 | -8 |
| van Haren [65] | 500ml of 6% HES in 0.9% saline over 15 minutes. | Isotonic bolus | 240 minutes | Mean | -200 ml/min/m2 | 3 | 0 | 3 |  | -0.2 | -40 | -4 |
| Dong [39] | 500ml of 6% HES over 30 minutes | Responders: > 15% SVI increase | 0 minutes. | Mean | +600 ml/min/m2 | -1.5 | 15.2 | 3.2 |  |  |  |  |
| Dong [39] | 500ml of 6% HES over 30 minutes | Non-responders: < 15% SVI increase | 0 minutes. | Mean | +300 ml/min/m2 | -1.2 | 4.8 | 2.3 |  |  |  |  |
| Khwannimit [45] | 500ml of 6% HES over 30 minutes | Responders: >15% SVI increase | 0 minutes. | Mean | +1300 ml/min/m2 | -3.3 | 9.5 | 3.4 |  |  |  |  |
| Khwannimit [45] | 500ml of 6% HES over 30 minutes | Non-responders: <15% SVI increase | 0 minutes. | Mean | +200 ml/min/m2 | -0.9 | 3.9 | 5.2 |  |  |  |  |
| Lakhal [46] | 500ml of 4% gelatin over 30 minutes | Responders: >15% SVI increase | 0 minutes. | Mean | +900 ml/min/m2 | -6 | 14 | 3 |  |  |  |  |
| Lakhal [46] | 500ml of 4% gelatin over 30 minutes | Non-responders: <15% SVI increase | 0 minutes. | Mean | +0 ml/min/m2 | -3 | 7 | 4.5 |  |  |  |  |
| Freitas [40] | 7ml/kg, max 500ml, of 6% HES over 30 minutes | Responders: > 15% CO increase | 30 minutes | Mean | +2100 ml/min | -2 | 11 | 3 | 8% | -0.1 |  |  |
| Freitas [40] | 7ml/kg, max 500ml, of 6% HES over 30 minutes | Non-responders: < 15% increase in CO | 30 minutes | Mean | +200 ml/min | 0 | 8 | 5 | -3.50% | -0.2 |  |  |
| **FIRST AUTHOR** | **Fluid Given** | **Group** | **Time from completion of fluid administration until physiological measurement** | **Measure of Central Tendency** | **Change in Cardiac Output Estimation** | **Change in heart rate (bpm )** | **Change in Mean Arterial Pressure (mmHg))** | **Change in central venous pressure (mmHg)** | **Change in venous oxygen saturation (%)** | **Change in blood lactate concentration (mmol/l)** | **Change in urine output** | **Change in haemoglobin concentration (g/L)** |
| **Colloid fluid boluses (contd)** | | | | | | | | | | | | |
| Wacharasint [66] | 500ml of 6% HES over 30 minutes | All patients | 30 minutes | Mean | +470 ml/min/m2 | 0.3 | 9.2 | 5.25 |  |  |  |  |
| **Mixed and undifferentiated fluid boluses** | | | | | | | | | | | | |
| Pottecher [60] | Up to 500ml of 6% HES or 0.9% saline over 30 minutes | All patients | 30 minutes | Mean | +1400 ml/min | -2 | 7 |  |  |  |  |  |
| Ospina-Tascon [57] | 400ml of 4% albumin or 1000ml of CSL over 30 minutes | Patients with early sepsis | 60 minutes | Median | +300 ml/min/m2 | 2 | 2 | 3 | 2% | -0.2 |  |  |
| Ospina-Tascon [57] | 400ml of 4% albumin or 1000ml of CSL over 30 minutes | Patients with late sepsis | 60 minutes | Median | +300 ml/min/m2 | -9 | 7 | 1 | 1% | 0.1 |  |  |
| Pierrakos [59] | 500ml of 6% HES or 1000ml of CSL over 30 minutes | Responders: > 10% increase in CI. | 30 minutes | Mean | +600 ml/min/m2 | -4 | 8 | 3 | 3% |  |  |  |
| Pierrakos [59] | 500ml of 6% HES or 1000ml of CSL over 30 minutes | Non-responders: < 10% increase in CI. | 30 minutes | Mean | +0 ml/min/m2 | -4 | 3 | 2 | 0% |  |  |  |
| Bihari [36] | 500 - 750ml of 4% albumin, blood, 20% albumin FFP, 0.9% saline, 4% gelatin or platelts administered over less than 30 minutes. | All patients. | 60 minutes | Median |  | 0 | 2 | 2 | 0.40% | -0.2 | No change | -6 |
| SVC: superior vena cava; HES: hydroxyethyl starch; CSL: compound sodium lactate; SV: stroke volume; SVI stroke volume index; CO: cardiac output; CI: cardiac index; VO2: oxygen delivery; | | | | | | | | | | | | |
